# X-ray microcomputed tomography applied to the taxonomic study of rare material: redescriptions of seven of Schirch’s Brazilian species of land planarians (Geoplanidae, Platyhelminthes)

**DOI:** 10.3897/zookeys.910.39486

**Published:** 2020-02-10

**Authors:** Marcos Santos Silva, Fernando Carbayo

**Affiliations:** 1 Laboratório de Ecologia e Evolução, Escola de Artes, Ciências e Humanidades, Av. Arlindo Bettio, 1000, Universidade de São Paulo, São Paulo, SP, Brazil; 2 Programa de Pós-Graduação em Zoologia, Instituto de Biociências, Rua do Matão, Travessa 14, Universidade de São Paulo, São Paulo, SP, Brazil; 3 Programa de Pós-Graduação em Sistemática, Taxonomia Animal e Biodiversidade, Museu de Zoologia, Universidade de São Paulo, São Paulo, SP, Brazil

**Keywords:** Brazil, classification, morphology, nomenclature, *
Pseudogeoplana
*, Tricladida

## Abstract

In 2016, the type-material of ten of the 15 Brazilian land planarians (Platyhelminthes, Tricladida, Geoplanidae) described by Schirch (1929) was discovered deposited in the Museu Nacional do Rio de Janeiro (MNRJ). Schirch only described the external morphology of these species, all originally placed in the genus *Geoplana*. By the 1930s and 1950s *Geoplana itatiayana*, *G. plana*, and *G. rezendei* underwent taxonomic revision based on the study of non-type specimens. The remaining 12 species also underwent a taxonomic revision but only based on the literature. Current names of these species are *Geoplana goettei*, *Pseudogeoplana arpi*, *Ps. blaseri*, *Ps. bonita*, *Ps. bresslaui*, *Ps. cardosi*, *Ps. doederleini*, *Ps. lumbricoides*, *Ps. obscura*, *Ps. riedeli*, *Ps. theresopolitana*, and *Ps. wetzeli*. The species *Geoplana maximiliani* sensu Schirch (1929) was renamed as *Ps. schirchi* Ogren & Kawakatsu, 1990.

The present study reports a taxonomic revision of seven of Schirch’s species using type material, namely *Obama itatiayana*, *Pasipha plana*, *Pseudogeoplana arpi*, *Ps. bresslaui*, *Ps. doederleini*, *Ps. schirchi* and *Ps. wetzeli*. Additional specimens of some of these species were also examined. Morphological data from histological preparations and from virtual sections were obtained through a non-destructive technique of X-ray computed microtomography (µCT). This approach resulted in the preservation of the entire body of at least one type-specimen of each species, and the holotype of *Ps. bresslaui*. Conspecificity of *O. itatiayana* and *P. plana* was confirmed, as previously reported in the literature. It is also proposed that *Ps. bresslaui* belongs to the genus *Paraba*, while the other species should remain in *Pseudogeoplana*, since type-specimens are either immature, poorly preserved or simply lost.

## Introduction

Land planarians (Geoplanidae, Tricladida, Platyhelminthes) are free-living predators most diversified in intertropical forests. Approximately 910 species are currently known (Sluys 2016), a quarter of them occurring in Brazil (Almeida et al. 2019).

Almost all Neotropical land planarians belong to the monophyletic subfamily Geoplaninae Stimpson, 1857 (Carbayo et al. 2013). The current 25 genera of this subfamily are diagnosed by aspects related to the distribution of sensory pits, organization of the cutaneous and parenchymal musculature, the morphology of the pharynx and, more importantly, the copulatory apparatus. The collective group *Pseudogeoplana* Ogren & Kawakatsu, 1990 houses species of the Geoplaninae which are insufficiently known, especially regarding their copulatory apparatus, due to either the immaturity of specimens or the absence of histological sections (Ogren and Kawakatsu 1990).

Schirch (1929) described 15 new Brazilian species, which he placed in the genus *Geoplana* Stimpson, 1857 (Geoplaninae), viz., *Geoplana arpi*, *G. blaseri*, *G. bonita*, *G. bresslaui*, *G. cardosi*, *G. doederleini*, *G. goettei*, *G. itatiayana*, *G. lumbricoides*, *G. obscura*, *G. plana*, *G. rezendei*, *G. riedeli*, *G. theresopolitana*, and *G. wetzeli*. All descriptions were exclusively based on external characters, such as the size, shape, and color of the body and the distribution of eyes and their halos (small pigment-free areas of the dorsum encircling eyes). Type material of Schirch’s species has never been reexamined.

Later, four of these species were redescribed from new material: *G. goettei*, *G. itatiayana*, *G. plana*, and *G. rezendei* (Riester 1938, Marcus 1951, Froehlich 1955a). Currently, these four species are now classified in different genera, as *Paraba goettei*, *Obama itatiayana*, *Pasipha plana*, and *Issoca rezendei* (Froehlich 1955a, Carbayo et al. 2013). The remaining eleven species are placed in *Pseudogeoplana*, viz., *Ps. arpi*, *Ps. blaseri*, *Ps. bonita*, *Ps. bresslaui*, *Ps. cardosi*, *Ps. doederleini*, *Ps. lumbricoides*, *Ps. obscura*, *Ps. riedeli*, *Ps. theresopolitana*, and *Ps. wetzeli*.

Schirch lost the type material of *Ps. cardosi*, *G. goettei*, and *Ps. riedeli* (Schirch 1929), but he did not mention whether type specimens of the remaining 12 species were deposited in any museum. Marcus was the first scientist resident in Brazil to resume studies on the Brazilian land planarian fauna (Carbayo et al. 2009). From him, successors learned that Schirch’s type specimens were either lost or were so poorly conserved that there would be no point in studying them (E. M. Froehlich, pers. comm.).

Recently, we inquired of the Brazilian Museu Nacional do Rio de Janeiro (MNRJ) concerning Schirch’s specimens and, contrary to expectations, were informed that the types of ten of Schirch’s species were housed in the museum, viz., *Issoca rezendei*, *Obama itatiayana*, *Pasipha plana*, *Pseudogeoplana arpi*, *Ps. blaseri*, *Ps. bonita*, *Ps. bresslaui*, *Ps. doederleini*, *Ps. schirchi*, and *Ps. wetzeli*. Fortunately, we received the material on loan prior to the catastrophic fire in the MNRJ.

In this paper, we undertake a taxonomic revision of *O. itatiayana*, *P. plana*, *Ps. arpi*, *Ps. bresslaui*, *Ps. doederleini*, *Ps. schirchi*, *Ps. wetzeli*, and the putative *Ps. blaseri* by studying the type material and, where available, additional specimens. Revisions of *Issoca rezendei* and *Ps. bonita* will be reported elsewhere. Whenever possible, we studied the types using microcomputed tomography-derived (µCT) images, so that the physical integrity of the specimens would be preserved (see Faulwetter et al. 2013, Laforsch et al. 2012, Molineux et al. 2007). We also tried to overcome the limitations regarding the resolution of µCT-derived images, as encountered in a previous taxonomic study of another land planarian (Carbayo et al. 2016, Carbayo and Lenihan 2016).

## Materials and methods

All syntypes presumably studied by Schirch (1929), available in the MNRJ, were studied. We received the specimens wrapped in a paper towel moistened with 80 % ethanol inside 5–10 ml plastic vials. Upon receipt of the material, we filled the plastic tubes with 80 % ethanol. Each syntype was given an additional label to distinguish it from the remaining type specimens. The holotype of *Ps. doederleini* and one of the syntypes of *Ps. schirchi* showed signs of having been accidentally dehydrated (body roughened and rigid) and, therefore, these were immersed in Sandison’s solution (Sandison 1955) for five days for tissue rehydration prior to µCT or histological processing. We also examined additional specimens of *Pasipha plana*, in particular histological slides of one specimen studied by E. M. Froehlich (1955a) and one specimen from the wet collection of F. Carbayo which was sectioned for examination.

### X-ray microcomputed tomography (µCT)

The largest syntype or the holotype (by monotypy, as designated below) of each of Schirch’s species were processed for µCT (Table 1). These specimens were immersed in a solution of 0.3 % phosphotungstic acid (PTA) and 3 % dimethyl sulfoxide (DMSO) in 95 % ethanol for at least 30 days on a laboratory rocker to facilitate PTA penetration into the body (Fernández et al. 2014). Subsequently, they were rinsed in 95 % ethanol and transferred to a vial with 95 % ethanol for µCT data acquisition. Specimens which were scanned twice were submerged again into PTA solution one week before the next scan. The entire body of each specimen was scanned at low resolution to get information on the gross internal morphology as well as its state of maturity. Specimens with a relatively narrow body were scanned at higher resolutions. Only the holotype of *Ps. bresslaui* was mature and, because its body is relatively narrow, it was scanned at almost the highest resolution provided by the equipment. Therefore, we attempted to describe the holotype entirely from the µCT dataset.

**Table 1.** Availability of type material of Schirch’s (1929) species. * Type specimens studied in this work. ^+^Additional non-type specimens also studied.

Original name	Current name	Taxonomic revisions from conspecific specimens	Number of type specimens according to Schirch (1929)	Number of type specimens deposited in the MNRJ (reference)
*Geoplana arpi*	*Pseudogeoplana arpi*	No	A few specimens	2*
*G. blaseri*	*Ps. blaseri*	No	1	Lost (this work)
*G. bonita*	*Ps. bonita*	No	Not detailed	7
*G. bresslaui*	*Ps. bresslaui*	No	1	1*
*G. cardosi*	*Ps. cardosi*	No	1	Lost prior to deposition (Schirch 1929)
*G. doederleini*	*Ps. doederleini*	No	Not detailed	3*
*G. goettei*	*G. goettei*	Froehlich (1955a)	1	Lost prior to deposition (Schirch 1929)
*G. itatiayana*	*Obama itatiayana*	Riester (1938); Carbayo et al. (2013)	Several	3*^+^
*G. lumbricoides*	*Ps. lumbricoides*	No	Not detailed	Lost (this work)
*G. maximiliani*	*Ps. schirchi*	No	1	1*
*G. obscura*	*Ps. obscura*	No	1	Lost (this work)
*G. plana*	*Pasipha plana*	Froehlich (1955a)	Not detailed; apparently some	4*^+^
*G. rezendei*	*Issoca rezendei*	Marcus (1951); Froehlich (1955a)	Two at least	2
*G. riedeli*	*Ps. riedeli*	No	1	Lost prior to deposition (Schirch 1929)
*G. theresopolitana*	*Ps. theresopolitana*	No	1	Lost (Carbayo and Almeida 2015)
*G. wetzeli*	*Ps. wetzeli*	No	Some	7*

Two scanners were used. A General Electric V-Tomex scanner (housed in the Laboratory of Paleontologia e Microtomografia, Museu de Zoologia da Universidade de São Paulo) was used for scanning the type material of *Obama itatiayana*, *Paraba bresslaui*, *Pasipha plana*, *Pseudogeoplana arpi* (two scans), *Ps. blaseri*, *Ps. doederleini*, *Ps. schirchi*, and *Ps. wetzeli* specimens under the basic settings ranging from 25–75 kV, and 150–4000 mA. Three scans of the holotype of *Ps. bresslaui* were run using a Zeiss Xradia Versa XRM-510 scanner (Laboratório de Caracterização Tecnológica (LCT) da Escola Politécnica da Universidade de São Paulo) (Table 2).

Visualization, editing, and manipulation of the virtual volumes and reconstructed microtomographic images were done using the programs myVGL (https://www.volumegraphics.com/en/products/myvgl.html) and DataViewer (http://bruker-microct.com/products/downloads.htm). 3D images were generated with CTVox software (http://www.skyscan.be/next/CTvox64.zip).

**Table 2.** Type specimens and their body regions scanned using X-ray micro-computed tomography.

Scan no.	Species	Specimen	Body region	Medium voltage (kV)	Current (mA)	Image pixel size (µm)	Scan duration in hrs: mins (equipment)
1	*Obama itatiayana*	Syntype C	Entire body	70	220	7.3	25 m (GE V-Tomex)
2	*O. itatiayana*	Syntype C	Pharynx and Copulatory apparatus	70	220	6.3	25 m (GE V-Tomex)
3	*Paraba bresslaui*	Holotype	Cephalic extremity	50	4000	1.9	04 h:00 m (Xradia versa XRM - 510)
4	*Pa. bresslaui*	Holotype	Copulatory apparatus	50	4000	4.5	01 h:40 m (Xradia versa XRM - 510)
5	*Pa. bresslaui*	Holotype	Prostatic vesicle	50	4000	1.9	04 h:00 m (Xradia versa XRM - 510)
6	*Pa. bresslaui*	Holotype	Entire body	25	180	7.99	59 m (GE V-Tomex)
7	*Pa. bresslaui*	Holotype	Copulatory apparatus	70	310	2.95	34m (GE V-Tomex)
8	*Pasipha plana*	Syntype A	Entire body	55	250	12	0 h:42 m (GE V-Tomex)
9	*Pseudogeoplana arpi*	Syntype A	Entire body	75	290	17.01	34 m (GE V-Tomex)
10	*Ps. arpi*	Syntype A	Pharynx	75	240	10	34 m (GE V-Tomex)
11	*Dolichoplana*sp.	Non-type	Entire body	70	280	11.92	25 m (GE V-Tomex)
12	*Ps. doederleini*	Syntype A	Entire body	60	350	5	34 m (GE V-Tomex)
13	*Ps. schirchi*	Holotype	Pharynx	70	150	9	25 m (GE V-Tomex)
14	*Ps. wetzeli*	Syntype A	Entire body	70	310	4.77	34 m (GE V-Tomex)

### Histology

Fixed specimens were examined and photographed using an Olympus SZ61 stereo-microscope and a Leica M80 stereo-microscope with a Leica IC80HD digital camera attached to it. Specimens were divided into several portions, each of which was histologically processed as follows: dehydration in alcohol series, de-alcoholization in clove oil, inclusion in Paraplast, serial sectioning at intervals of 5–7 µm with a rotary microtome (Microm HM315 R), rehydration and, subsequent, staining with Mallory’s trichrome, as modified by Cason (1950). Distribution of the eyes was observed in specimens cleared in clove oil. Histological slides were examined and photographed using an Olympus BX51 optical microscope and a DP72 digital camera attached to it. Drawings of the pharynx and copulatory apparatus were made by using a camera lucida attached to the microscope. Nomenclature of body color follows RAL CLASSIC colors (https://www.ral-farben.de/content/application-help/all-ral-colours-names/overview-ral-classic-colours.html). Whenever possible, all figures are orientated so that the anterior extremity of the body is to the left.

### Abbreviations

**ab** annular-shaped muscle bands

**cg** cerebral ganglion

**cm** circular muscle

**cmc** common muscular coat

**cn** cutaneous nerve net

**co** common glandular ovovitelline duct

**cs** creeping sole

**de** dorsal epithelium

**dd** decussate diagonal fiber

**dm** decussate muscle fiber

**e** eye

**ef** epithelium of female atrium

**ej** ejaculatory duct

**es** esophagus

**f** fold

**fa** female atrium

**g** gonopore

**gl** glands

**i** intestine

**lc** longitudinal cutaneous muscle

**lm** longitudinal muscle

**lp** longitudinal parenchymal muscle

**m** muscle

**ma** male genital atrium

**mo** mouth

**ns** nervous system

**o** ovary

**ov** ovovitelline duct

**ph** pharyngeal pouch

**pp** penis papilla

**pi** sensory pit

**pv** prostatic vesicle

**sb** subintestinal transverse parenchymal muscle

**sd** sperm duct

**sg** shell glands

**sp** supraintestinal transverse parenchymal muscle

**t** testis

**ve** ventral epithelium

**vi** vitellaria

**vn** ventral nerve plate

## Taxonomic section

### Obama itatiayana

Taxon classificationAnimaliaTricladidaGeoplanidae

(Schirch, 1929)

905A5893-E638-56F4-94BE-F750238802AE

Figures 1 , 2 , 3

Geoplana itatiayana Schirch, 1929: 34. Type locality: Teresópolis, State of Rio de Janeiro, Brazil.
Geoplana itatiayana : Riester 1938: 64–66.
Obama itatiayana : Carbayo et al. 2013: 523. comb. nov.

#### Material examined.

Type material. Teresópolis, State of Rio de Janeiro, Brazil. P. Schirch *Coll* 1916. Each syntype was given an additional identification with a letter, A-C. **MNRJ 216A**: Body preserved in 80 % ethanol. **MNRJ 216B**: Body preserved in 80 % ethanol. **MNRJ 216C**: Three dimensional (3D) images and virtual sections were obtained by microcomputed tomography. Transverse sections of anterior extremity on 27 slides; horizontal sections of ovaries region on 39 slides; transverse sections of pre-pharyngeal region on 28 slides; sagittal sections of pharynx region on 111 slides and sagittal sections of copulatory apparatus region on 77 slides. Remaining part of body preserved in 80 % ethanol.

#### Diagnosis.

Species of *Obama* ~43–53 mm long; dorsal color traffic yellow, interspersed with pearl gold specks; esophagus 16 % of pharynx length; prostatic vesicle of inverted-J shape, with an intrabulbar portion; male atrium measuring 4 % of body length and twice as long as female atrium; penis papilla tapered, shorter than male atrium; female atrium funnel-shaped, 1.4 % body length.

#### External aspect.

**Figure 1.**
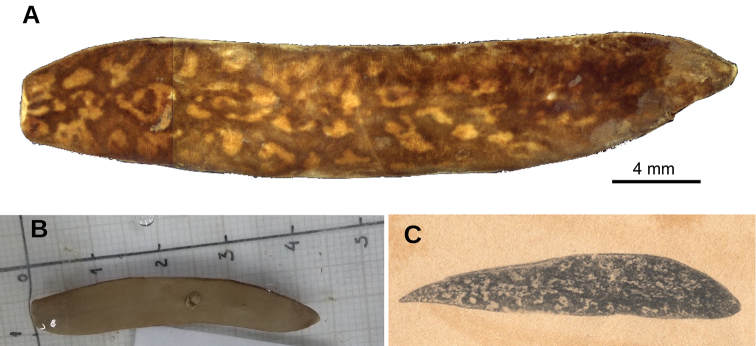
*Obama itatiayana* (Schirch 1929), syntype C **A** dorsal view **B** ventral view **C** original drawing by Schirch (1929).

Fixed, syntype C measured ~43 mm long, 7 mm wide and 1.6 mm high. Elongated body, with margins parallel; anterior end approximately rounded, posterior tapering (Fig. 1A). Dorsum convex, ventral side flat. Ground color of dorsum traffic yellow interspersed with pearl gold specks, thus resembling ocellated color of ocelot *Leopardus pardalis*. Ventral side lemon yellow (Fig. 1B).

The monolobate eyes are distributed on the anterior tip of the body, encircling the apex of the anterior tip. Posteriorly, the eyes spread onto the dorsum to occupy a band on either side of the body, with a maximum of 22 % of the body width, near region of the ovaries; towards posterior extremity they become less numerous. Sensory pits are simple invaginations 40 µm deep, located ventro-marginally in a single row that surrounds anterior extremity and continues posteriorly up to at least 13 mm of body length, as measured from anterior margin. Relative position mouth: body length, 56 %; relative position gonopore, 75 % in syntype C.

#### Internal morphology.

**Figure 2.**
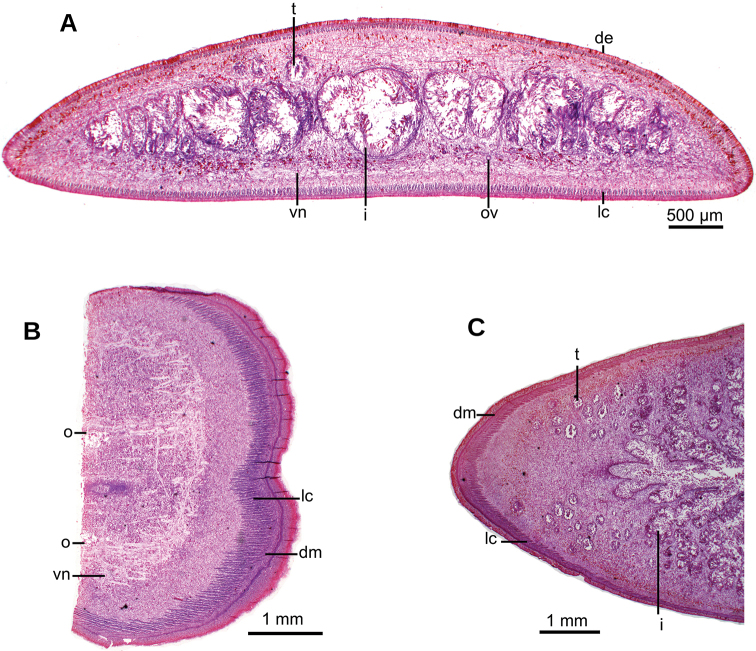
*Obama itatiayana* (Schirch 1929). Syntype C **A** photomicrograph of a transverse section of pre-pharyngeal region **B** photomicrograph of horizontal section of ovarian region **C** photomicrograph of horizontal section of testicular region.

Creeping sole comprising 93 % of body width. Abundant rhabditogen cells and glands producing erythrophil granules pierce dorsal and marginal epidermis; glands producing amorphous orangish-to-reddish secretion pierce marginal epidermis. Glandular margin is composed of glands producing erythrophil granules. Cutaneous musculature comprises three layers, viz., a subepithelial circular layer, followed by two diagonal layers with decussate fibers, and then a well-developed innermost layer of longitudinal muscles. Muscle fibers of longitudinal layer (45 µm thick dorsally; 75 µm thick ventrally) are arranged into bundles with 23–78 fibers each. Cutaneous musculature thickness relative to body height in pre-pharyngeal region, 10 % (Fig. 2A, C). Three parenchymal muscle layers, i.e. a dorsal layer of decussate diagonal fibers, a supraintestinal layer of transverse fibers, and a subintestinal layer with transverse fibers. Ventral nerve plate present (Fig. 2A, B).

**Figure 3.**
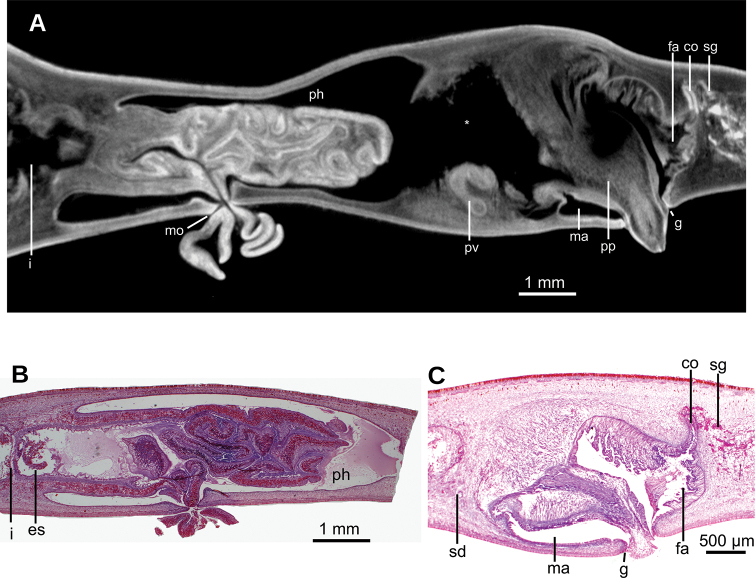
*Obama itatiayana* (Schirch 1929). Syntype C **A**µCT-derived image. Virtual sagittal section of pharynx and copulatory region, pixel size: 6.3 µm **B** photomicrograph of sagittal section of pharynx **C** photomicrograph of sagittal section of copulatory apparatus.

Mouth situated at a distance from root of pharynx equivalent to 40 % of pharyngeal pocket length. Pharynx bell-shaped. Esophagus 500 µm in length (Fig. 3A, B). Outer pharyngeal musculature consists of one-fiber-thick layer of longitudinal muscle, followed by a layer of circular muscle (15 µm thick) with longitudinal fibers interspersed. Inner pharynx musculature consisting of a subepithelial layer of circular fibers (50 µm thick) interspersed with longitudinal fibers.

Testes ~250 µm in diameter; the follicles are dorsally located between the dorsal parenchymal muscle layer and supraintestinal parenchymal layer (Fig. 2C). Anteriormost testes at a distance from the anterior extremity of the body equivalent to 11 % of body length; posteriormost testes are anterior to the pharynx, and at a distance equivalent to 46 % of body length. Efferent ducts run between the oviducts and dorsally to the nerve plate. Efferent ducts communicate with the latero-proximal region of the prostatic vesicle (Fig. 3C). Prostatic vesicle divided into an anterior, extrabulbar half running dorsally, and a posterior, intrabulbar half having the shape of an inverted J (Fig. 3A). Anterior half of prostatic vesicle with folded wall and lined by a 50 µm high columnar ciliated epithelium which is pierced by glands producing erythrophil granules. Intrabulbar half of vesicle lined with a 30 µm high epithelium; this intrabulbar half pierced by glands producing erythrophil granules being more intensely stained than those in the anterior half. Prostatic vesicle surrounded by a 50 µm thick layer of muscle fibers variously oriented, mainly, diagonal and circular. Prostatic vesicle continues as ejaculatory duct within the penis papilla. This duct curves ventrally to open at the tip of the penis papilla. Ejaculatory duct lined with an erythrophil, 5 µm high ciliated epithelium and surrounded by a circular muscle layer (12 µm thick). Penis papilla conical, with dorsal insertion shifted posteriorly and lined with an 80 µm high columnar epithelium, which is pierced by glands producing erythrophil granules. This epithelium is surrounded by a 100 µm thick coat of muscles consisting of interlaced circular and longitudinal fibers (Fig. 3C).

Male atrium 1.7 mm long (4 % of body length), not folded, partially occupied by the penis papilla. Male atrium lined with a 15 µm high non-folded columnar epithelium, which is pierced by the openings of abundant glands producing erythrophil granules (Fig. 3A, C). Male atrium covered by a 12 µm thick layer of circular and diagonal muscle fibers, best visible in its ventral region.

Ovaries ovoid, ~400 µm in maximum length, situated above the ventral nerve plate (Fig. 2B), situated at a distance from anterior extremity equivalent to 30 % of the body length. Ovovitelline ducts emerge from the lateral portion of the ovaries. Anterior to the gonopore region, the oviducts curve dorso-medially and, subsequently, join to form a common glandular ovovitelline duct, the latter located dorsally to the female atrium (Fig. 3C). Shell glands pierce the distal portion of the ovovitelline ducts. Common glandular ovovitelline duct 300 µm long and running ventrally to enter the female genital canal. Common ovovitelline duct and female genital canal lined by a ciliated epithelium, which is surrounded by a 10 µm thick layer of circular muscle (Fig. 3A, C).

Female atrium 750 µm long, funnel-shaped, with its posterio-dorsal portion receiving the female genital canal. Female atrium lined with a 50–400 µm high epithelium; this epithelium presents a stratified aspect and its free surface is indented (Fig. 3A, C). Female atrial epithelium is pierced by the openings of abundant glands, producing erythrophil granules, and surrounded by a weak muscle layer of circular fibers that is contiguous with that on the common muscle coat. Male atrium: female atrium ratio, 2:1.

Common muscle coat consists of interwoven circular and longitudinal muscle fibers enveloping the unpaired portion of the prostatic vesicle, male and female atria, and part of the common glandular ovovitelline duct.

#### Distribution.

Municipality of Teresópolis (-22.42, -43.01) and Itatiaia (-22.41, -44.65), State of Rio de Janeiro, Brazil.

#### Remarks.

The three syntypes A, B, C, and individuals studied by Riester (1938) are very similar to each other in their external aspect. Syntype C and Riester’s specimens are also similar in their internal organs, namely the shape of the pharynx and the copulatory apparatus.

Riester (1938) redescribed the species from additional specimens. Froehlich (1955b) synonymized *Geoplana duca* Marcus, 1951, from São Paulo and Mogi das Cruzes (state of São Paulo) with *G. itatiayana*. However, body color and details of the male and female atria of *G. duca* do not match those of *O. itatiayana*. In this regard, we disagree with Froehlich’s taxonomic action. A revision of the taxonomic status of *G. duca* is, therefore, desirable.

**Figure 4.**
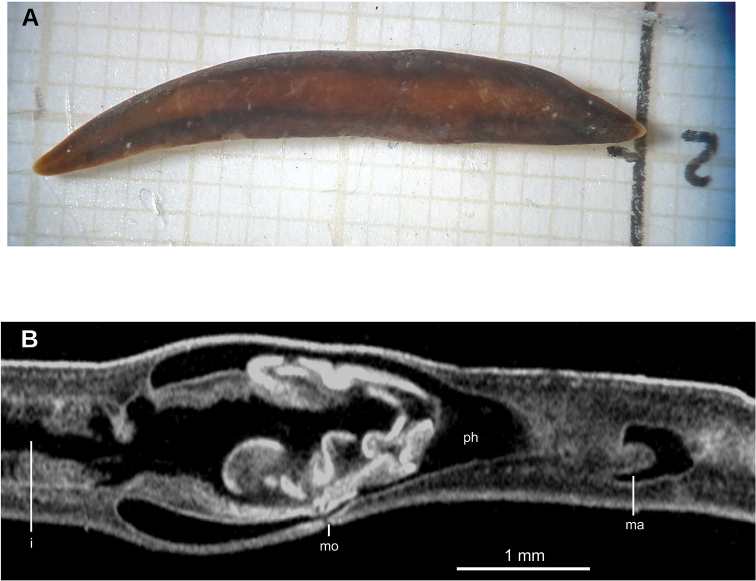
Specimen MNRJ 8906 mislabeled as *O. itatiayana***A** in dorsal view **B** virtual sagittal section of pharynx and incipient copulatory apparatus, pixel size: 10 µm.

A fourth putative type specimen of *O. itatiayana*, viz., specimen MNRJ 8906, does not conform to the original description as its body is narrower, the dorsum more convex and has a pearl orange midstripe and a pair of paramedian pale brown stripes, external to which the color is beige gray (Fig. 4A). The pharynx and copulatory apparatus of this specimen (Fig. 4B), as revealed from µCT-derived virtual sections, resemble *O. itatiayana*, but the specimen is incompletely mature. The vial of this specimen also contains a label reading “*Geoplana itatiayanensis*”, as well as another reading “*G. itatiayana*”. We suspect that at some point during curatorial handling this individual was mislabeled and confounded with the types of *O. itatiayana*.

### Paraba bresslaui

Taxon classificationAnimaliaTricladidaGeoplanidae

(Schirch, 1929) comb. nov.

D83DB09D-14CC-52D0-9E2E-72F5586B2F52

**Figure 5.**
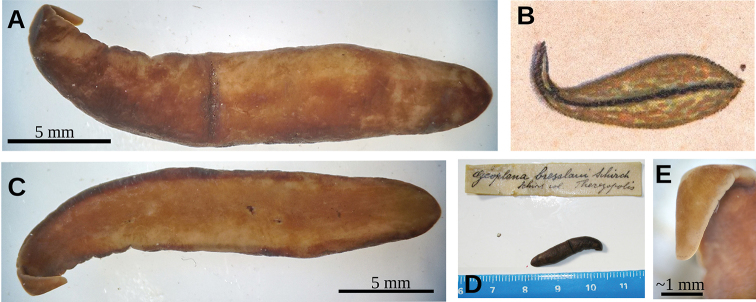
*Paraba bresslaui* (Schirch 1929), holotype **A** dorsal view **B** original drawing by Schirch (1929)**C** ventral view **D** dorsal view with original label **E** anterior extremity.

**Figure 6.**
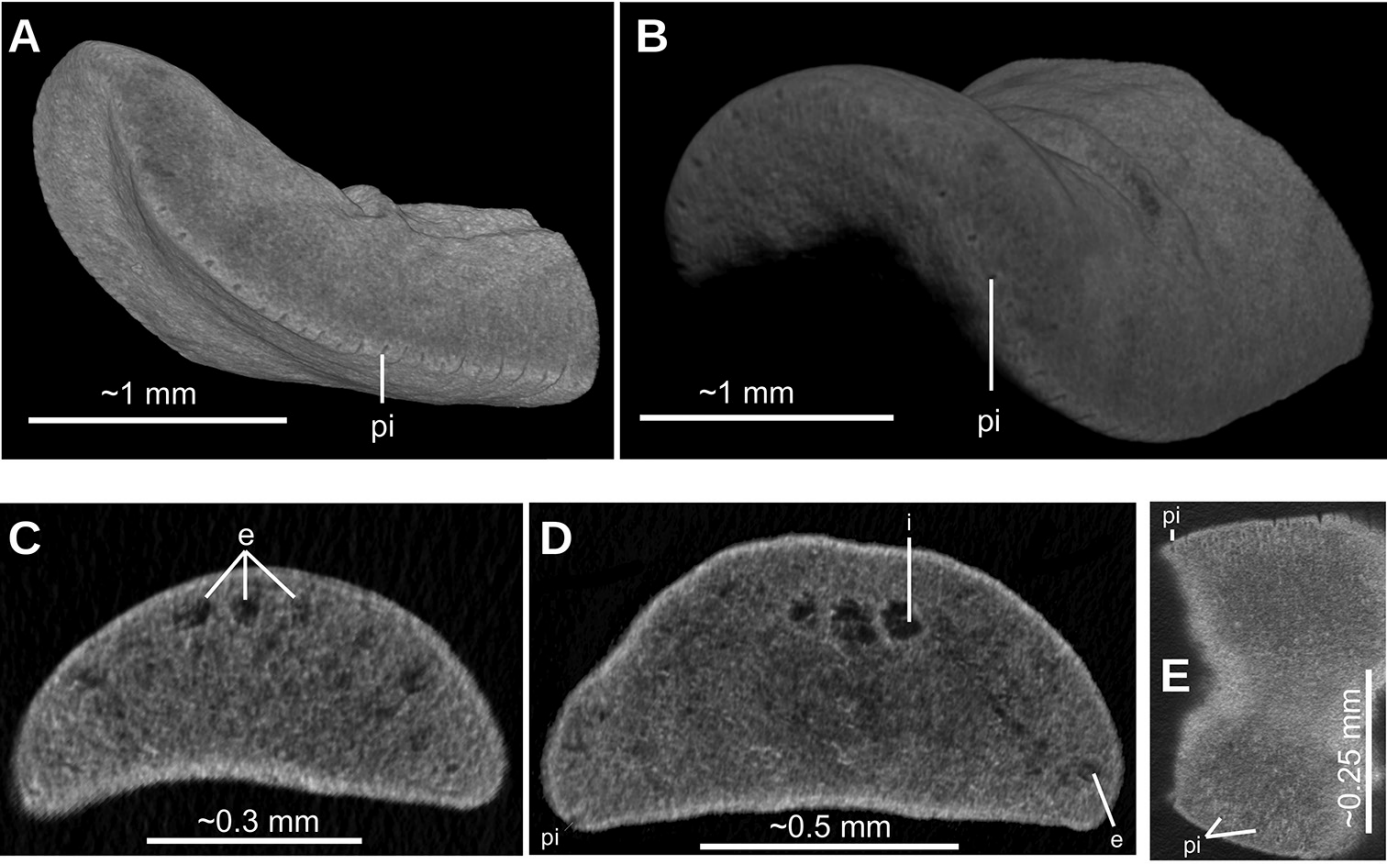
*Paraba bresslaui* (Schirch 1929), µCT-derived images of cephalic extremity of holotype **A, B** perspective view of 3D rendering **C** virtual transverse section showing anteriormost eyes, pixel size: 1.9 µm **D** virtual transverse section showing eye and sensory pit, pixel size: 1.9 µm **E** horizontal virtual section showing rows of sensory pits, pixel size: 1.9 µm.

**Figure 7.**
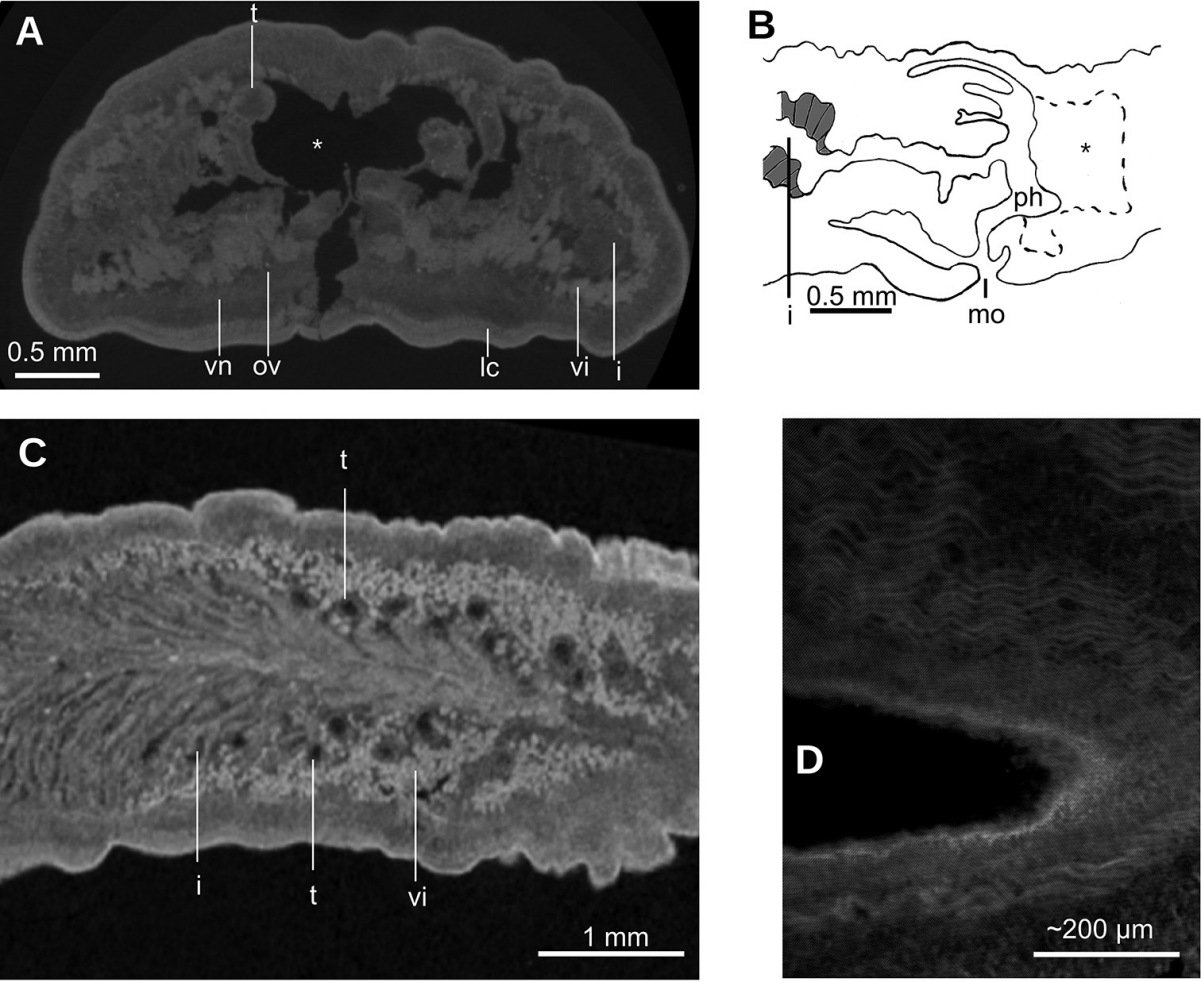
*Paraba bresslaui* (Schirch 1929), holotype. µCT-derived images and diagrammatic reconstruction **A** virtual transverse section of pre-pharyngeal region, pixel size: 7.99 µm **B** diagrammatic reconstruction of sagittal sections of pharynx **C** virtual horizontal section of testicular region, pixel size: 7.99 µm **D** virtual horizontal section of copulatory region, showing tangential section of ventral cutaneous muscle fibers, pixel size: 4.5 µm. Asterisk indicates artifact.

**Figure 8.**
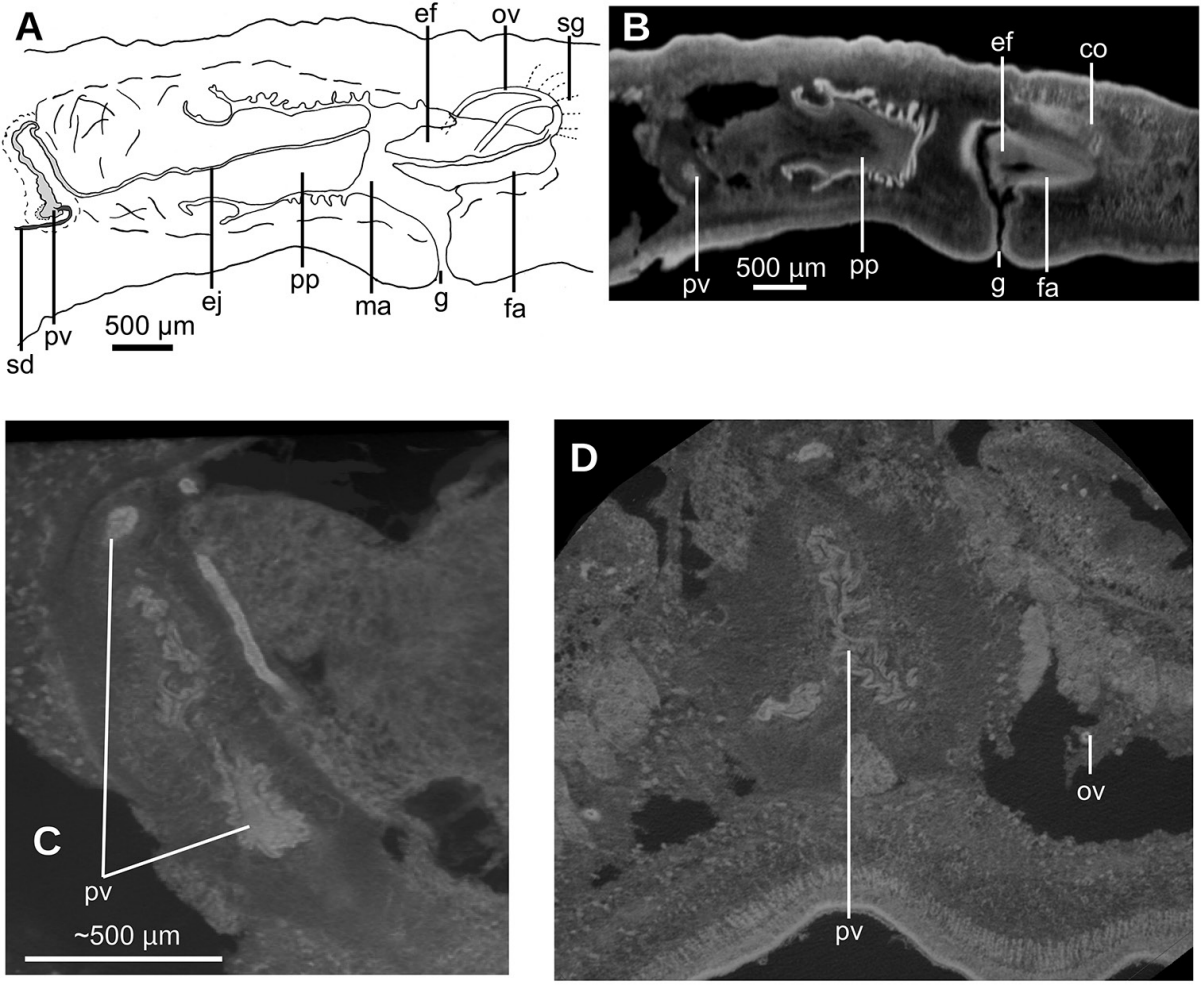
*Paraba bresslaui* (Schirch 1929), holotype **A** diagrammatic reconstruction of copulatory apparatus **B** virtual sagittal section of copulatory apparatus, pixel size: 4.5 µm **C** virtual sagittal section showing prostatic vesicle, pixel size: 1.9 µm **D** virtual transverse section of body showing prostatic vesicle, pixel size: 1.9 µm.

**Figure 9.**
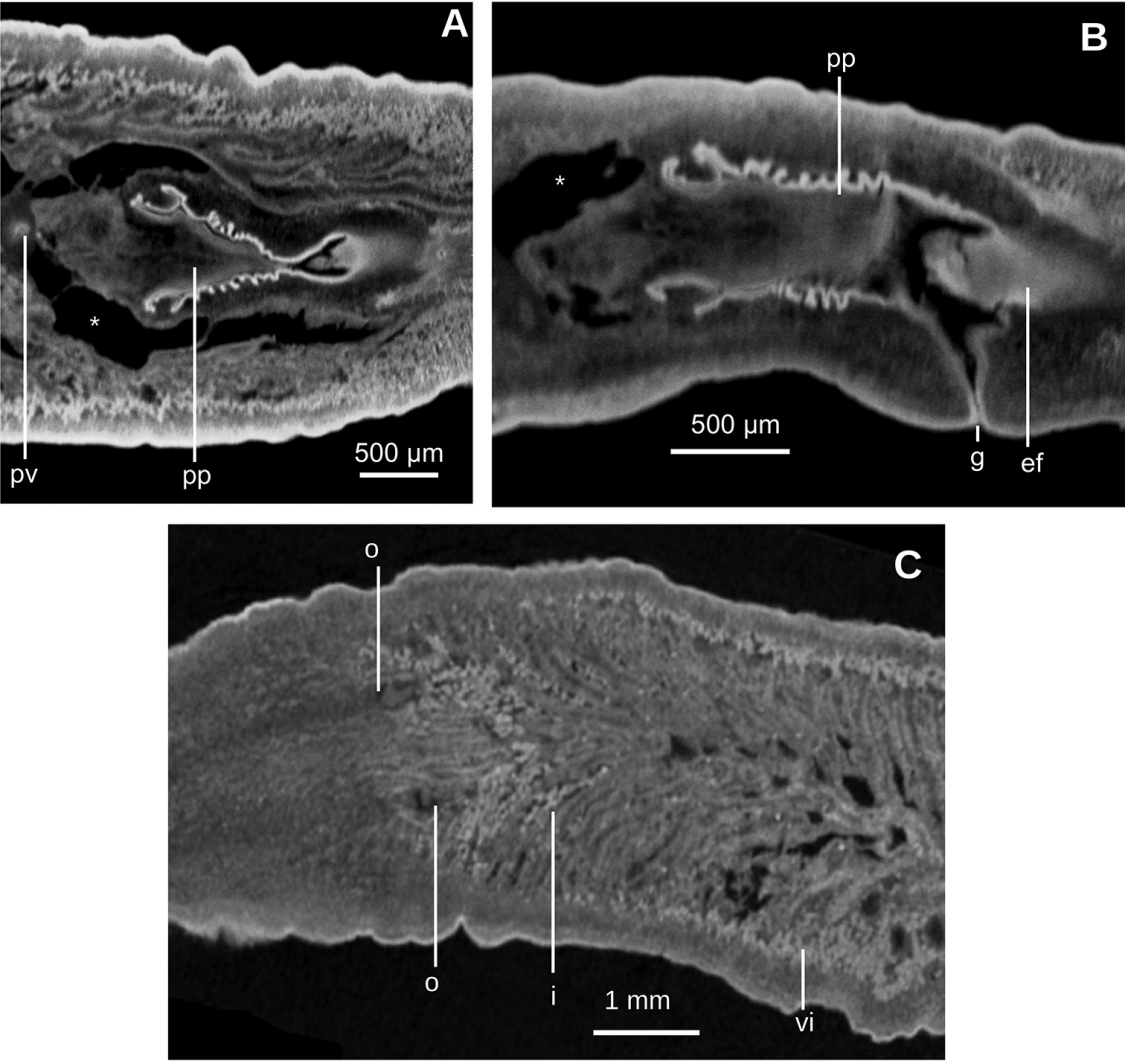
*Paraba bresslaui* (Schirch 1929), holotype **A** virtual horizontal section of copulatory apparatus, pixel size: 7.99 µm **B** virtual sagittal section of copulatory apparatus, pixel size: 7.99 µm **C** horizontal section of ovarian region, pixel size: 7.99 µm.

Figures 5 , 6 , 7 , 8 , 9

Geoplana bresslaui Schirch, 1929: 31. Type Locality: Teresópolis, State of Rio de Janeiro, Brazil.
E. M. Froehlich 1955b: 203. [nec Geoplana bresslaui Schirch, 1929, in Riester (1938)].
Pseudogeoplana bresslaui : Ogren and Kawakatsu 1990: 114.

#### Material examined.

**MNRJ 210, holotype**, here designated by monotypy: Teresópolis, State of Rio de Janeiro, Brazil. P. Schirch (unknown year). Preserved in 80 % ethanol. Three dimensional (3D) images and virtual sections were obtained by microcomputed tomography.

#### Diagnosis.

Species of *Paraba* 25 mm long; dorsal side yellowish green ground color with a narrow median black band; long and narrow prostatic vesicle, inverted ‘U’-shape; male atrium with 6–8 transverse annular folds; ovovitelline ducts ascend anterior to gonopore; female atrium lined with an epithelium that protrudes into male atrium.

#### External aspect.

Fixed holotype 25 mm long; body with greatest width in the pharyngeal region, 5 mm. Towards the extremities, the body narrows gently, with the anterior tip being rounded and the posterior end being obtuse (Fig. 5A, C). Dorsal side strongly convex; ventral side flat. Dorsum ochre brown, with irregular areas of cream color as a result of fading (Fig. 5A, D). Body margins in anterior 1/6 of the body with beige color. Ventral side ochre yellow (Fig. 5C).

A single row of eyes contours the anterior first millimeter of the body (Fig. 5E); posteriorly they extend for ca. 4 mm, arranged in a row with 2–4 eyes on either side of the body. Further posterior, eyes could not be discerned. Sensory pits are simple invaginations located ventro-marginally in a single row from the very anterior body tip to at least 2 mm posteriorly (Fig. 6A, E). Relative position mouth: body length, 66 %. Relative position gonopore: body length, 82 %.

#### Internal morphology.

Two-millimeter long paired cerebral ganglia, commencing 5 mm posterior to anterior extremity. Ventral nerve plate present (Fig. 7A). Longitudinal cutaneous musculature well developed, with fibers gathered in bundles (Fig. 7A, D). Cutaneous musculature thickness relative to body height at the pre-pharyngeal region, ~4 %. Cephalic retractor muscle absent.

Mouth opening at the posterior section of the pharyngeal pouch. Pharynx cylindrical (Fig. 7B, C), with a dorsal insertion slightly posterior to the ventral one. Short esophagus present. Testes rounded, the largest follicles ca. 200 µm in diameter. They are located dorsally and are arranged in a row of ca. 20 follicles on either side of the body (Fig. 7D), each row having up to two testes in the same transverse plane. Most anterior testes are located dorsally to the ovaries and 1 mm posterior to the cerebral ganglia, i.e., at a distance from anterior extremity equivalent to 32 % of the body length; posterior-most testes shortly anterior to the pharyngeal root.

Lateral to the prostatic vesicle, the sperm ducts recurve and then open into a branch of the prostatic vesicle (Fig. 8D). Prostatic vesicle with an inverted ‘U’-shape in lateral view (Fig. 8C), with its ascending portion having a sinuous wall, while the descending portion is straight. At its distal most portion, this vesicle bends posteriorly to penetrate the antero-ventral region of the penis bulb. Prostatic vesicle surrounded by a 100 µm thick distinct mass, presumably consisting of muscles and glands. This vesicle is located at anterior portion of the penis bulb, the latter extending 1 mm anterior to the penis papilla.

Inside the penis bulb, the prostatic vesicle communicates with the ejaculatory duct. This duct is horizontally positioned and runs through the penis papilla to open at its tip. Penis papilla cylindrical, with the distal portion truncated in lateral view (Figs 8A, B, 9A, B), but conical in dorsal view (Fig. 9A). It fills 2/3^rd^ of the male atrium length. Male atrium broad, with 6–8 transverse, annular folds. Anterior-most fold is the largest and surrounds the basis of the penis papilla. Length of male atrium twice of that of the female atrium.

Ovaries 230 µm in diameter and approximately rounded. They are positioned at 8 mm from anterior extremity of the body, and 1 mm posterior to the cerebral ganglia. They are located above the ventral nerve plate (Fig. 7D). Ovovitelline ducts emerge from the dorso-lateral aspect of the ovaries (Fig. 7A) and run posteriorly horizontally to the level of the gonopore. Then they ascend obliquely to the sagittal plane and join the common glandular ovovitelline duct immediately above the posterior region of the female atrium. Common glandular ovovitelline duct (~100 µm long) runs ventrally to join the female genital canal, the latter being a ~160 µm projection of the posterio-dorsal region of the female atrium. Female atrium ~1 mm in length and 0.5 mm in height, equivalent of 1/4 of the body height. This female atrium is elongate and is lined with an epithelium with a very high epithelium (~250 µm), typical of epithelia with a stratified aspect, which occupies the whole cavity except for an irregular narrow canal (Fig. 9A, B). This female lining epithelium presents a cone-shaped protrusion into the male atrium. The gonopore canal is vertical and 500 µm long.

#### Distribution.

Municipality of Teresópolis (-22.42, -43.01), State of Rio de Janeiro, Brazil.

#### Remarks.

The general shape and size of the body agrees with the original description (“length 25 mm; maximum width 8–9 m[m]” (Schirch 1929) and correct labeling of the specimen as the holotype by monotypy is assumed (Fig. 5B). Fading of the body color is normal, especially after 90 years of conservation in a preservative.

The specimen has the characteristics of the subfamily Geoplaninae, albeit the width of the creeping sole could not be ascertained. We assume a wide creeping sole on the basis of the flat ventral side of the specimen.

Further features observed on this species match only one genus of the Geoplaninae, namely *Paraba*Carbayo et al., 2013, whose diagnosis reads: “Geoplaninae with small-to-medium-sized body, 6–80 mm in length; body slender, with margins nearly parallel; dorsum and ventral side slightly convex; eyes monolobulated; pharynx cylindrical; prostatic vesicle extrabulbar, generally horizontal; penis papilla protrusible, conical; male atrium not folded; ascending portion of ovovitelline ducts lateral to the gonopore canal or to female atrium, joining each other above female atrium; genital canal dorso-anteriorly flexed, arising from the posterior-dorsal region of female atrium; female atrium rounded, clothed with an epithelium with multilayered aspect” (Carbayo et al. 2013: 523). In view of the internal morphology of the species, herein presented for the first time, it is justifiable to transfer the species to *Paraba*.

This species only differs from the diagnostic features of *Paraba* in some details that deserve a comment: (a) the dorsum is not slightly, but strongly convex, (b) the male atrium is not unfolded, but folded, and (c) the female atrium is not rounded, but elongate. These differences probably represent artefacts or intrageneric variation: the two former features (shape of the dorsum and folds of the male atrium) might have been caused by contraction during fixation or, simply, the male atrium is folded. An elongate female atrium is also found in other species of *Paraba* (namely *Pa. franciscana*, *Pa. rubidolineata*).

Regarding the external aspect, *Pa. bresslaui* differs from all other congeneric species in the color pattern, consisting of a yellowish green ground color, ornamented with a median black stripe, and beige pigment spots, often elongated and parallel to the main body axis. None of the 14 species of *Paraba* present a green-yellowish dorsal color with a median black stripe and beige spots stretching longitudinally, as in *Pa. bresslaui*.

Regarding the internal aspect, there are four species resembling *Pa. bresslaui* in having a U-shaped prostatic vesicle, namely *Pa. cassula* (E. M. Froehlich, 1955a), *Pa. goettei* (Schirch, 1929), *Pa. phocaica* (Marcus, 1951) and *Pa. piriana* (Almeida & Carbayo, 2012, in Almeida et al. 2012). However, *Pa. phocaica* is the only species sharing with *Ps. bresslaui* the posterior insertion of the genital canal into the female atrium. Both species also share distinct features: an annular fold around the basis of the penis papilla, a male atrium folded, and the general shape of the prostatic vesicle. Apart from body color, however, *Pa. bresslaui* is distinguished from *Pa. phocaica* in that the latter species presents a more dorsal entrance of the prostatic vesicle into the penis bulb (vs. ventral as in *Pa. bresslaui*), a rounded female atrium (vs. elongate), and a lining epithelium of the female atrium not invading the male atrium (vs. invading it).

### Pasipha plana

Taxon classificationAnimaliaTricladidaGeoplanidae

(Schirch, 1929)

D041B515-F0F6-5B8F-9191-AD2C6B61769C

**Figure 10.**
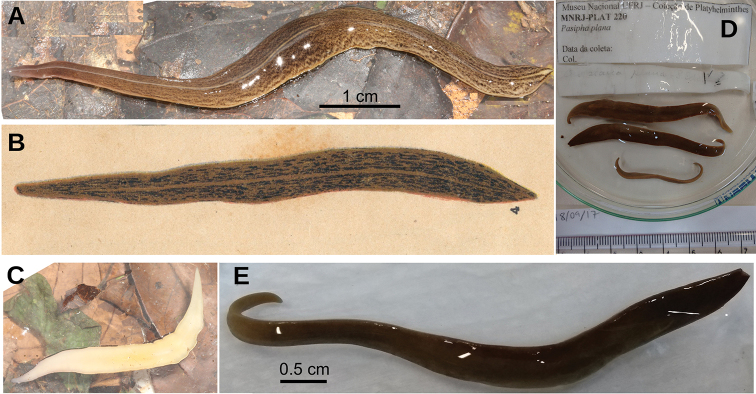
*Pasipha plana* (Schirch 1929) **A** live specimen F6484 in dorsal view **B** original drawing by Schirch (1929)**C** ventral view of live specimen F6484 **D** set of all syntypes with anterior extremity to right **E** syntype B fixed in dorsal view.

**Figure 11.**
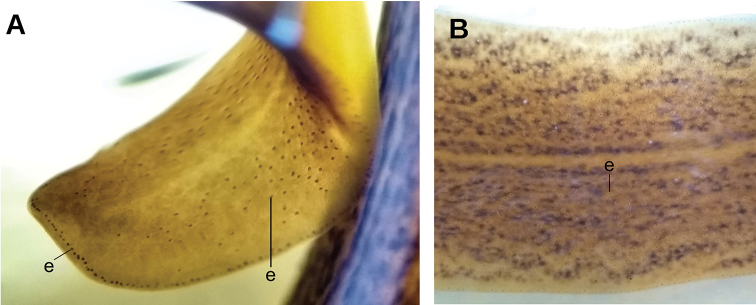
*Pasipha plana* (Schirch 1929), specimen F6484 in clove oil **A** dorsal anterior extremity **B** dorsal mid-body region.

**Figure 12.**
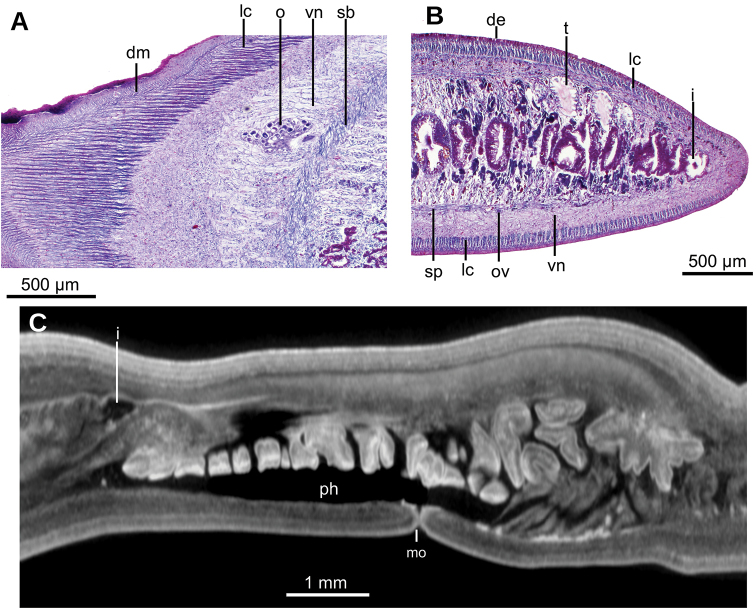
*Pasipha plana* (Schirch 1929) **A** specimen F6484 photomicrograph of a horizontal section of ovaries region **B** F6484 photomicrograph of transverse section of pre-pharyngeal region **C** syntype A virtual section of a sagittal section of pharynx, pixel size: 12 µm.

**Figure 13.**
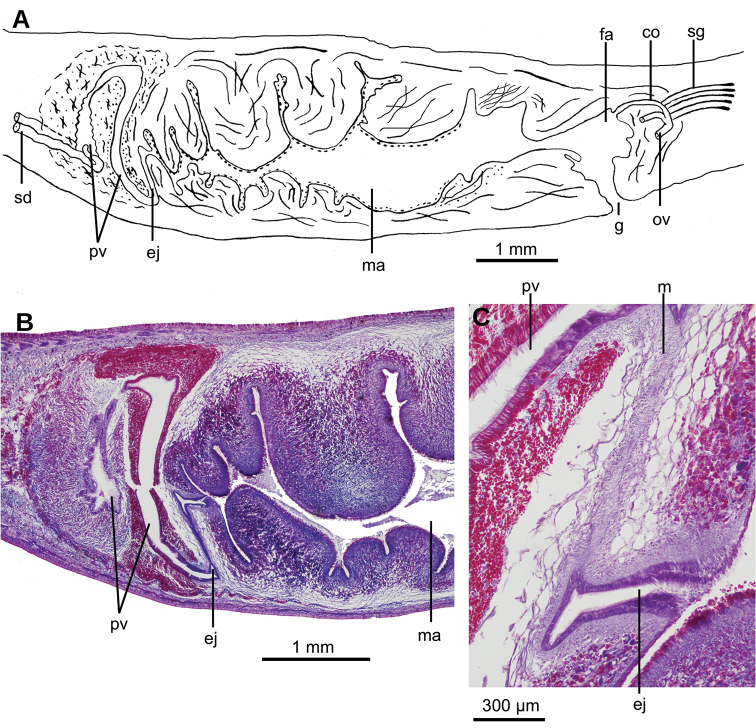
*Pasipha plana* (Schirch 1929) **A** specimen F6484 diagrammatic reconstruction of copulatory apparatus **B** photomicrograph of prostatic vesicle, ejaculatory duct, and male atrium in sagittal section **C** photomicrograph of distal portion of prostatic vesicle and of ejaculatory duct in sagittal section.

**Figure 14.**
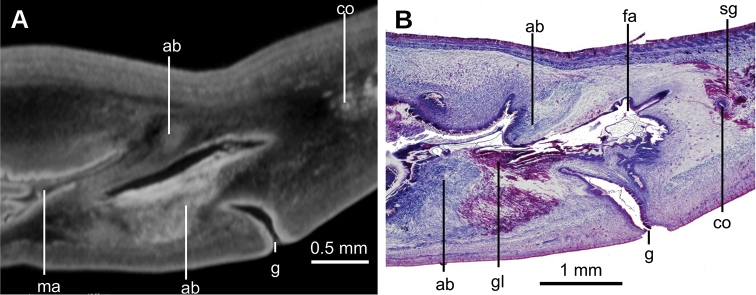
*Pasipha plana* (Schirch 1929). Specimen F6484 **A** virtual sagittal section of copulatory apparatus, pixel size: 12 µm **B** photomicrograph of sagittal section of copulatory apparatus.

**Figure 15.**
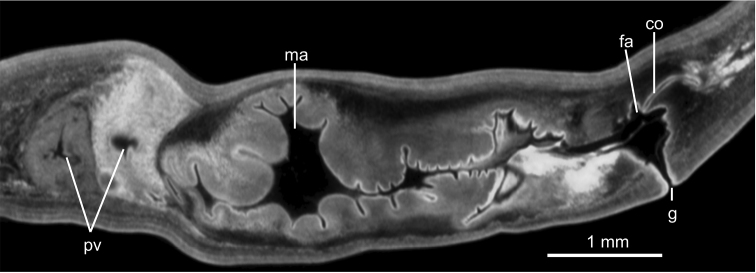
*Pasipha plana* (Schirch 1929). Syntype A virtual sagittal section of copulatory apparatus, pixel size: 12 µm.

Figures 10 , 11 , 12 , 13 , 14 , 15

Geoplana plana Schirch, 1929: 33. Type Locality: Teresópolis, Rio de Janeiro, Brazil.
Geoplana plana : E. M. Froehlich 1955a: 299.
Pasipha plana : Ogren and Kawakatsu 1990: 51.

#### Material examined.

Type material. Four syntypes collected by P. Schirch *Coll* (year unknown) in Teresópolis, State of Rio de Janeiro, Brazil. We received them on loan in 70 % ethanol with only a label reading 8915. Each syntype was given an additional identification with a letter, A–D. Three dimensional (3D) images and virtual sections of syntype MNRJ 220A were obtained by microcomputed tomography. Parts of the bodies of syntypes were histologically sectioned as follows. Syntype **MNRJ 220A**: Transverse sections of posterior extremity on 14 slides; remaining part of body preserved in 80 % ethanol. Syntype **MNRJ 220B**: Sagittal sections of copulatory apparatus on 156 slides; remaining part of body preserved in 80 % ethanol. Syntype **MNRJ 220C**: Horizontal sections of anterior extremity on 31 slides; horizontal sections of ovaries on 71 slides; transverse sections of pre-pharyngeal region on 35 slides; sagittal sections of pharynx on 94 slides; sagittal sections of copulatory apparatus on 124 slides; remaining part of body preserved in 80 % ethanol. Syntype **MNRJ 220D**: Preserved in 80 % ethanol.

Additional material examined: Specimen **MZUSP PL** XXXX, studied by E. M. Froehlich (1955a): Teresópolis, Parque Nacional da Serra dos Órgãos, State of Rio de Janeiro, Brazil (geographic coordinates not available), E. M. Froehlich et al., leg. 1953. Transverse sections of pre-pharyngeal region on two slides (S761-S762); sagittal sections of pharynx on one slide (S763); sagittal sections of copulatory apparatus on ten slides (S764-S773). **F6484** (MZUSP PL XXXX): Parque Estadual da Pedra Branca, Rio de Janeiro, State of Rio de Janeiro, Brazil, -22.933, -43.447, Carbayo et al., leg. 9 Dec. 2014. Sagittal sections of copulatory apparatus on 101 slides; remaining part of body preserved in 80 % ethanol.

#### Diagnosis.

Species of *Pasipha* 60–70 mm long; dorsum with black brown dots on ivory ground color; eyes dorsal, occupying lateral band on each side of body, 44 % of body width; posteriormost testes between pharynx and copulatory apparatus; prostatic vesicle inverted U-shaped in lateral view, not bifurcated; ovovitelline ducts emerge from inner-dorsal side of ovaries; male atrium folded; length of male atrium to female atrium ratio, 8:1; female atrium as long as high.

#### External aspect.

Living adult (F6484) 70 mm in length and 10 mm wide. Fixed adults (F6484 and syntype C) 65–71 mm long, 6 mm wide, and 1.7 mm high. Elongated body, with margins approximately parallel; anterior and posterior extremities rounded. Dorsum slightly convex, ventral side flat. Ground color of the dorsum of living animals is ivory, richly ornamented with black brown dots (Fig. 10A, B, D, E). Towards the body margins, these dots tend to anastomose into longitudinal striae. Striae are lacking in a thin medial stripe which is bordered on each side by an irregular line of the same color as the dots. Ventral side is sulfur yellow, with the anterior extremity light ivory and the pharyngeal region zinc yellow (Fig. 10C). After 89 years or more in a preservative, the dorsal color of the four syntypes has turned ochre yellow and the dots faded to a terra brown color (Fig. 10D, E); their ventral body surface has become olive yellow.

Monolobate eyes surround the anterior extremity of the body (Fig. 11A). They also spread onto the entire dorsum except for a thin median line ~8 % of the body width (Fig. 11B). Towards the posterior extremity, they become scarcer. Sensory pits are simple invaginations 25 µm deep, located ventro-marginally in a single row from the very anterior body tip up to at least 57 % of the body length (syntype C). Relative position of the mouth 69 % of body length, that of the gonopore 90 % in syntype C (68 %, and 91 %, in specimen F6484).

#### Internal morphology.

Creeping sole comprising 96 % body width. Abundant rhabditogen cells and glands producing erythrophil granules pierce dorsal and marginal epidermis; glands producing amorphous orangish-to-reddish secretion pierce marginal epidermis. Ventral epithelium is pierced by glands producing fine erythrophil granules. Glandular margin absent. Cutaneous musculature comprises three layers, namely a subepithelial circular layer, followed by a double diagonal layer with decussate fibers, and then a well-developed, innermost longitudinal layer (Fig. 12A, B). Muscle fibers of the longitudinal layer (120 µm thick dorsally; 88 µm thick ventrally) are arranged into bundles with 30–50 fibers each. Cutaneous musculature thickness relative to body height at the pre-pharyngeal region, 13 %. Three parenchymal muscle layers present, all well-developed: dorsal layer of decussate diagonal fibers (60 µm thick), supraintestinal layer of transverse fibers (100 µm thick), and subintestinal layer with transverse fibers (65 µm thick; Fig. 12A, B). Ventral nerve plate present.

Mouth situated at a distance from the root of the pharynx equivalent to 50 % of the pharyngeal pocket length. Pharynx collar-shaped (Fig. 12C), with dorsal insertion very close to the end of the pharyngeal pocket. Esophagus absent. Outer pharyngeal musculature difficult to discern; apparently it consists of a one-fiber-thick subepithelial muscle layer (6 µm thick) of longitudinal fibers followed by an equally thick layer of circular fibers. Inner pharynx musculature consisting of a subepithelial layer of circular fibers (80 µm) in the anterior region of the pharynx; this circular layer is followed by scattered longitudinal fibers in the posterior region of the pharynx (syntype C).

Testes are ~300 µm in diameter and dorsally located between the supraintestinal parenchymal muscle layer and intestine. Anteriormost testes at a distance from the anterior extremity of the body equivalent to 29 % of body length; posteriormost testes are posterior to the pharynx, close to the prostatic vesicle, and at a distance equivalent to 85 % of the body length (syntype C).

Sperm ducts contain sperm in their distal region; a 60 µm thick circular muscle surrounds the distal region of these ducts. Sperm ducts run between the oviducts and onto the nerve plate. Lateral to the prostatic vesicle, efferent ducts curve forward and medially to communicate with the latero-proximal region of the prostatic vesicle (Fig. 13A).

Prostatic vesicle extrabulbar, long and narrow, divided into an anterior half running dorsally, and a posterior half, running ventrally, so describing an inverted U-course. Initial portion of prostatic vesicle with folded wall and lined by a 12–30 µm high cuboidal-to-columnar ciliated epithelium which is pierced by abundant glands producing erythrophil (purple) granules (Fig. 13B, C). Distal half lined with a 3 µm high epithelium that becomes 10 µm high in the section communicating with ejaculatory duct. This distal half pierced by numerous glands producing erythrophil (reddish) granules. Prostatic vesicle surrounded by variously oriented muscle fibers. This muscle is 200–400 µm thick around the proximal region, and thinner and less dense around distal region. Entire prostatic vesicle and its musculature are enveloped by a thin coat of muscle fibers, which are readily discerned from that of the common muscle coat.

Prostatic vesicle penetrates the ventral aspect of the common muscle coat and is continuous with the ejaculatory duct. This duct recurves dorsally before bending abruptly posteriorly to open into the anterior region of the male atrium (Fig. 13C). Ejaculatory duct lined with a cyanophil, 10–12 µm high, ciliated epithelium, and is surrounded by a circular muscle (10–15 µm thick) of thin fibers (1 µm in diameter).

Male atrium a 5 mm long cavity (syntype C, 8 % of body length) with large folds along its dorso-anterior 3/4. Posterior quarter is large but with smaller folds. Male atrium lined with an 8–15 µm high epithelium, ciliated along the anterior 3/4 of its length, and with its free surface papillate (Fig. 15). This epithelium is pierced by glands producing erythrophil (reddish-to-purple) granules and which are abundant throughout the entire epithelium, with the exception of that lining the sphincter (Fig. 14A, see below), which is pierced by abundant glands producing erythrophil (reddish) granules, especially in its ventral region (Fig. 14B).

Subepithelial musculature of male atrium consists of a 12 µm thick layer of circular and longitudinal muscle fibers, which sometimes intermingle and sometimes are arranged in two layers. This muscle continues with very abundant longitudinal and circular fibers located in the surrounding space delimited by the common muscle coat. A well-developed sphincter of circular muscle embraces the subterminal region of the male atrium. Dorsal region of the sphincter is posterior to the ventral (Fig. 14A, B).

Ovaries ovoid-shaped, 500 µm in maximum length, and situated immediately above the ventral nerve plate (Fig. 12A). They are at a distance from the anterior extremity equivalent to 22 % of the body length (syntype C). Ovovitelline ducts emerge from the inner-dorsal region of the ovaries. Posterior to the gonopore region, they curve medially and dorsally then join to form the common glandular ovovitelline duct, posteriorly to the female atrium. There are no shell glands discharging their secretion into the ovovitelline ducts (Fig. 13A). Common glandular ovovitelline duct 120 µm long (syntype C) and runs dorsally to enter the female genital canal. This canal (800 µm long in syntype C) is a projection of the posterio-dorsal region of the female atrium, and runs posteriorly and ventrally. Common ovovitelline duct and female genital canal are lined by a ciliated epithelium which is surrounded by a 25 µm thick muscle comprising fine (~1 µm) circular and diagonal fibers.

Female atrium short and ample, roughly as long as high. Its posterior wall projects anteriorly, giving it a square shape. Epithelial lining 12–25 µm high. Female atrial epithelium pierced by glands producing erythrophil granules and surrounded by a muscle layer of longitudinal and circular fibers in some regions, and deccussate in others. Male atrium: female atrium ratio, 8:1.

Common muscle coat is comprised of interwoven circular and longitudinal muscle fibers. It is less developed in its posterior region and envelopes the male and female atria and the common glandular ovovitelline duct (Figs 13A, 15).

#### Distribution.

Areas covered with Atlantic forest in the municipalities of Teresópolis (-22.42, -43.01) and Rio de Janeiro (-22.93, -43.44), State of Rio de Janeiro, Brazil.

#### Remarks.

Schirch (1929) described only the external appearance of this species. Froehlich collected one specimen in the type locality and described its external and internal morphology (E. Froehlich 1955a). By comparing her specimen with the succinct original description, she considered that they are conspecific (E. Froehlich 1955a). Indeed, apart from the external aspect, the internal morphology of Froehlich’s specimen matches in all aspects that of the syntypes, especially the copulatory apparatus of syntypes A, B, and C, herein described for the first time. This similarity also applies to the specimen F6484, recently collected by us. Thus, it is safe to assume that the syntypes, Froehlich’s specimen and specimen F6484 are all conspecific.

Currently, the species is placed in *Pasipha* Ogren & Kawakatsu, 1990. The diagnosis of this genus was revised by Carbayo et al. (2013). *Pasipha* comprises 24 species (plus five5 species considered *incertae sedis*, see Carbayo et al. 2013). Regarding the external aspect, *Pasipha plana* can be distinguished from all congeners in that it displays dark dots which anastomose into longitudinal striae.

Regarding the internal aspect, *Pa. plana* shares only with *Pa. cafusa* (Froehlich, 1956), *Pa. chimbeva* (E. M. Froehlich, 1955a), *Pa. oliveiroi* (Froehlich, 1955b), *Pa. pasipha* (Marcus, 1951), *Pa. penhana* (Riester, 1938), *Pa. pinima* (E. M. Froehlich, 1955a), *Pa. rosea* (E. M. Froehlich, 1955a), *Pa. splendida* (Graff, 1899), *Pa. tapetilla* (Marcus, 1951) and *Pa. velutina* (Riester, 1938) a non-branched prostatic vesicle. However, among these eleven species, only in *Pa. rosea*, *Pa. tapetilla* and *Pa. velutina* is the length of the male atrium to the female atrium ratio > 3:1, as in *Ps. plana*. In *Ps. plana*, however, this ratio is as high as 8:1. Furthermore, differing from *Ps. plana*, in *Pa. rosea* the prostatic vesicle is more sinuous and elongate, and the length of the female atrium is twice that of its height (vs. roughly as long as high in *Pa. plana*); in *Pa. tapetilla*, there is a barrel-shaped and muscular copulatory organ (vs. absent in *Pa. plana*). Finally, in *Pa. velutina* there is a penis papilla-like annular fold in the proximal region of the male atrium (vs. absent in *Pa. plana*).

### Pseudogeoplana arpi

Taxon classificationAnimaliaTricladidaGeoplanidae

(Schirch, 1929)

CF6914B7-F3F7-5705-9574-85CF0DC8C55F

**Figure 16.**
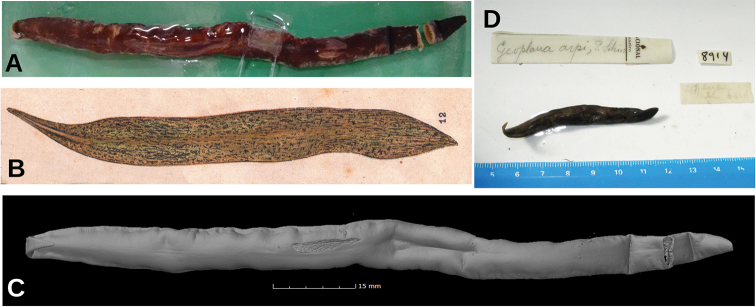
*Pseudogeoplana arpi* (Schirch 1929) **A** fixed syntype A in dorsal view **B** original illustration of *G. arpi* by Schirch (1929)**C** virtual µCT reconstruction of body of Syntype A **D** dorsal view of syntype B.

**Figure 17.**
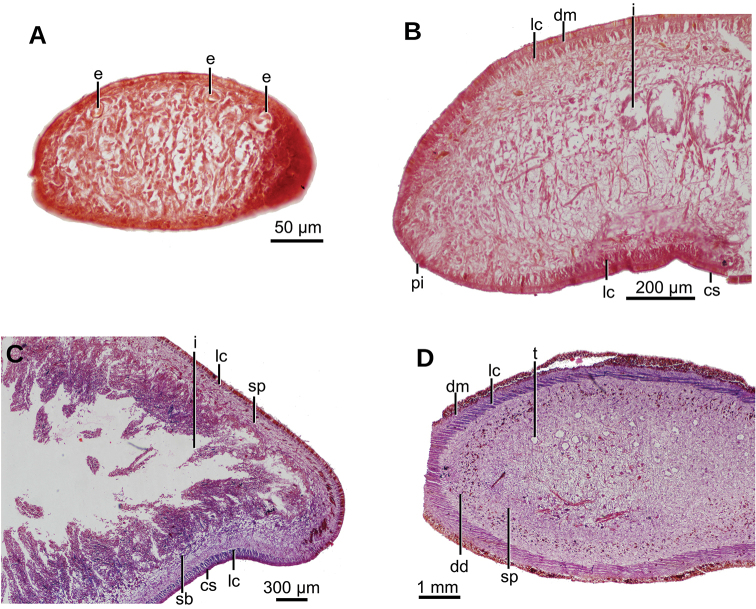
*Pseudogeoplana arpi* (Schirch 1929), photomicrographs of syntype B **A** transverse section of anterior extremity **B** transverse section of pre-pharyngeal region **C** transverse section of anterior extremity **D** horizontal section of testicular region.

**Figure 18.**
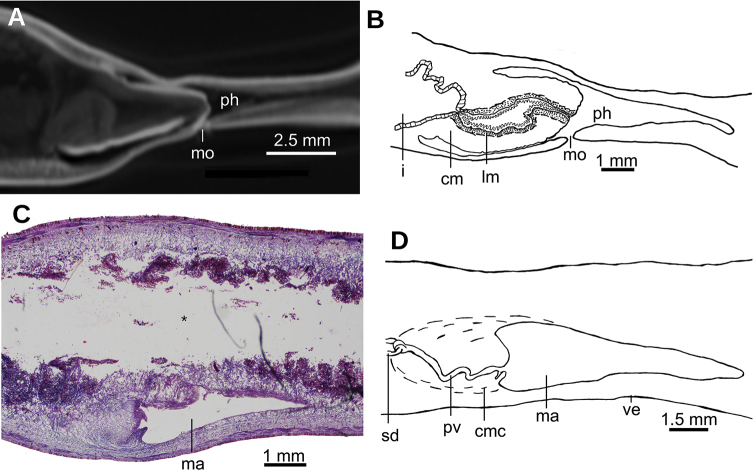
*Pseudogeoplana arpi* (Schirch 1929) **A** syntype A virtual sagittal section of pharynx, pixel size: 10 µm **B** syntype B diagrammatic reconstruction of sagittal sections of pharynx **C** syntype B photomicrograph of sagittal section of incipient copulatory apparatus **D** syntype B reconstruction of incipient copulatory apparatus.

Figures 16 , 17 , 18

Geoplana arpi Schirch, 1929: 33. Type Locality: Baixo Guandu, Espírito Santo, Brazil
Geoplana arpi : Ogren and Kawakatsu 1990: 113.
Pseudogeoplana arpi : Carbayo et al. 2013: 524.

#### Material examined.

Type material. Two syntypes available, collected by P. Schirch in 1917 at Baixo Guandu (former Maylasky), State of Espírito Santo, Brazil. We received them on loan in 70 % ethanol with label reading 8914. Three dimensional (3D) images and virtual sections of syntype **MNRJ 206A** were obtained by microcomputed tomography. Parts of body of syntype MNRJ 8914B were histologically sectioned as follows. Syntype **MNRJ 207B**: Transverse sections of anterior extremity on 20 slides; horizontal sections of a region posterior to anterior extremity, 4.5 mm long on 73 slides; transverse sections of pre-pharygeal region on 50 slides; sagittal sections of pharynx on 135 slides; remaining part of body preserved in 80 % ethanol.

#### External aspect.

Syntype 206 is 160 mm long, 10 mm wide, and ~2 mm high (Fig. 16A, C); syntype 207, 70 mm, ~8 mm, and ~2 mm (Fig. 16D), respectively. Elongated body, with margins approximately parallel; anterior extremity pointed, posterior rounded, body sides rounded. Dorsum convex, ventral side flat, convex on some portions of median region most probably the result of contraction at moment of fixation. Ground color of dorsum clay brown with irregular faded areas visible to naked eye (Fig. 16A, D). Ventral side grey beige.

Eyes monobolate, surround anterior extremity of the body (Fig. 17A) and are distributed evenly dorsolaterally along the entire body length. Sensory pits are simple invaginations 15 µm deep, located ventro-marginally in a single row (Fig. 17B) that contours the anterior extremity and extends posteriorly at least ~4 mm (or 7 % of the body length). Relative position of the mouth: body length, ~55 %. Relative position of incipient gonopore: body length, ~70 % in syntype 207.

#### Internal morphology.

Epithelium ciliated in anterior extremity, dorsally and ventrally; otherwise only ciliated on the ventral surface. Creeping sole comprising 92 % body width. In pre-pharyngeal region, abundant rhabditogen cells and glands producing erythrophil granules pierce dorsal and marginal epidermis; glands producing amorphous orangish-to-reddish secretion pierce marginal and dorsal epidermis. Ventral epithelium pierced by glands producing fine erythrophil granules. Glandular margin absent (Fig. 17C).

Cutaneous musculature comprises three layers, namely a subepithelial circular layer, followed by a diagonal layer of decussate fibers, and then a well-developed innermost longitudinal layer. Muscle fibers of the longitudinal layer (45 µm thick dorsally; 75 µm thick ventrally) arranged into bundles with 13–46 fibers each. Cutaneous musculature thickness relative to body height in the pre-pharyngeal region, 10 % (Fig. 17C). No muscle modifications in anterior extremity (Fig. 17A, B). Three parenchymal muscle layers present: a dorsal layer (10 µm thick) of decussate fibers, a supraintestinal layer (100 µm thick) with transverse fibers, and a poorly developed subintestinal layer (100 µm thick) of transverse fibers (Fig. 17D). Ventral nerve plate well developed.

Mouth situated at a distance from the root of the pharynx equivalent to 32 %-47 % of pharyngeal pocket length (syntypes 206 and 207, respectively). Pharynx cylindrical, tending to bell-shaped (Fig. 18A, B), occupying anterior half of the pharyngeal pocket. Esophagus absent. Free surface of outer pharyngeal epithelium undulated and ciliated, underlain by a subepithelial longitudinal muscle (8 µm thick) and followed by a circular muscle (50 µm thick). Inner pharyngeal musculature consisting of a subepithelial circular muscle (25 µm thick), followed by a longitudinal muscle (5 µm).

Testes poorly developed, rounded, ~100 µm in diameter, with no developed sperm. They are dorsally located among fibers of the supraintestinal transverse parenchymal muscle layer. Anteriormost testes at a distance from the anterior extremity of the body equivalent to 26 % of body length; posteriormost testes, 36 %, i.e., anterior to pharyngeal pocket (Fig. 17D).

Copulatory apparatus very poorly developed (Fig. 18C, D). It occupies the ventral 2/3^rd^ of the body height. Each of the paired sperm ducts opens into the proximal region of the bifurcate prostatic vesicle. This vesicle is intrabulbar and consists of an unpaired region running ventro-posteriorly inside a weakly developed penis bulb. Prostatic vesicle opens into a flat cavity, 35 µm high and 250 µm wide, delimited by a dorsal fold and a ventral fold. These folds project posteriorly from the anterior wall of the male atrium, forming a structure resembling a short, conical penis papilla. Ejaculatory duct not observed. Male atrium forms a 1.5 mm long closed cavity, i.e., it does not open to the outside of the body through an eventual gonopore canal. The atrium is narrower posteriorly and there is no differentiation between the male and female atria. The atrial epithelium is a mass of tissue, the limit of which is diffuse. Poor quality of the histological sections did not permit the description of additional details (Fig. 18C).

#### Remarks.

This species presents all the characteristics of the subfamily Geoplaninae, i.e., creeping sole covering most of the ventral surface, mouth posterior to the midbody, a well-developed cutaneous musculature organized into bundles, part of the parenchymal muscle layers organized in longitudinal fibers, and dorsal testes (see Almeida et al. 2019).

Since the syntypes present only poorly developed copulatory organs, it is not possible to allocate the species to any other genus. Consequently, the species should remain in *Pseudogeoplana*.

### Pseudogeoplana blaseri

Taxon classificationAnimaliaTricladidaGeoplanidae

(Schirch, 1929)

63637D8F-5AA3-5E5D-8007-AC2FA514907A

Figure 19

Geoplana blaseri Schirch, 1929. Type locality: unknown locality in the State of Espírito Santo, Brazil.
Geoplana blaseri : Ogren and Kawakatsu 1990: 114.
Pseudogeoplana blaseri : Carbayo et al. 2013: 524.

#### Remarks.

**Figure 19.**
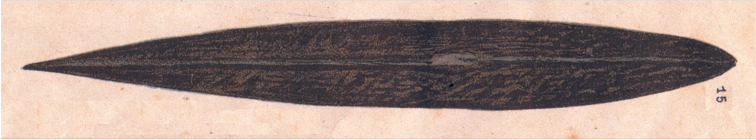
Original illustration of *G. blaseri* by Schirch (1929).

We received only one specimen identified as *Pseudogeoplana blaseri*. This specimen exhibits an aspect different from that of the original description. The external aspect was illustrated by Schirch (1929) (Fig. 19). In Schirch’s collection, no specimen resembles the original description of *Ps. blaseri*. A mislabeling must have happened prior to deposition or during the curation of the material. Thus, the holotype of *Ps. blaseri* should be considered lost. Nonetheless, we studied this available mislabeled specimen and provide further evidence of mislabeling (see “*Dolichoplana*sp.” in the following section).

### Rhynchodeminae Graff, 1896

#### Dolichoplana sp.

Taxon classificationAnimaliaTricladidaGeoplanidae

D076A79B-DE41-57FD-A5BE-8DD73F1D40DB

**Figure 20.**
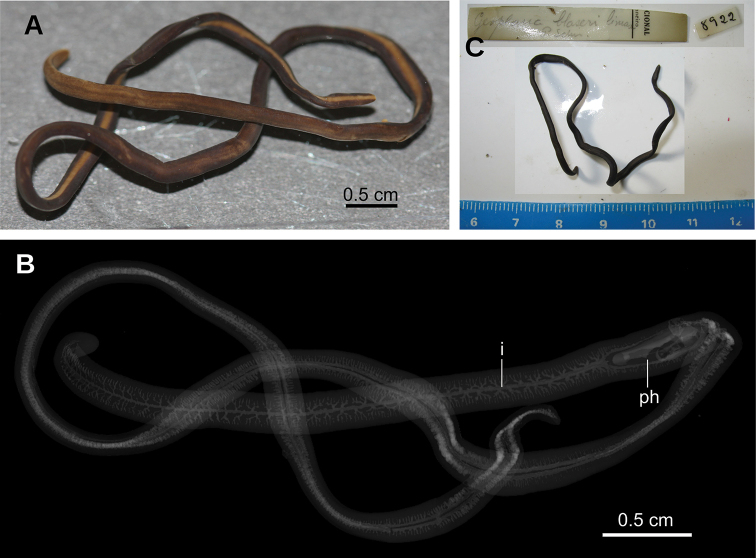
*Dolichoplana*sp. Specimen MNRJ 8922 **A** specimen fixed in dorsal view **B** volume rendering reconstruction of body from µCT data **C** fixed specimen with original label.

**Figure 21.**
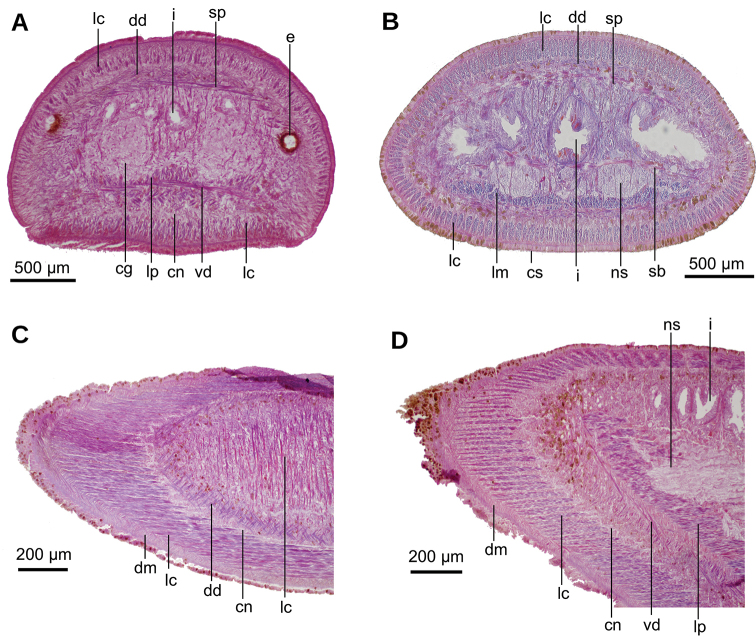
*Dolichoplana*sp. Photomicrographs of specimen MNRJ 8922 **A** transverse section of cephalic region **B** transverse section of pre-pharyngeal region **C** horizontal section of pre-pharyngeal region showing dorsal muscles **D** horizontal section of pre-pharyngeal region showing ventral muscles.

**Figure 22.**
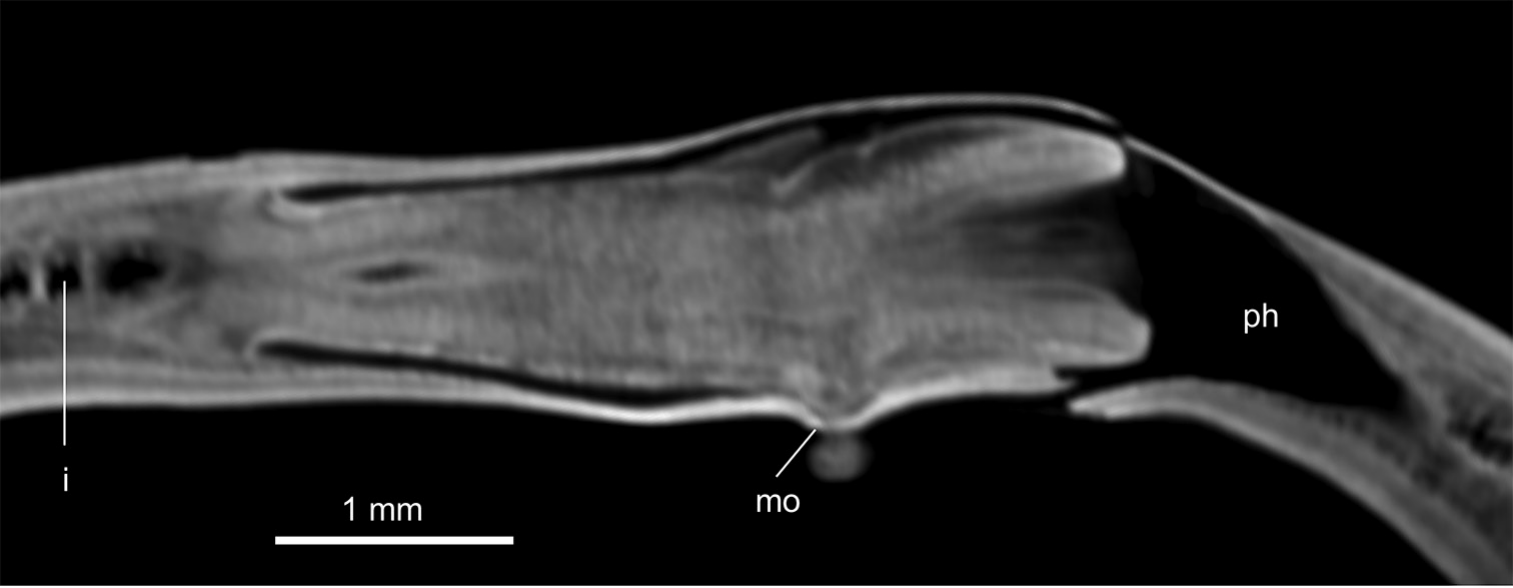
*Dolichoplana*sp. Specimen MNRJ 8922. Virtual sagittal section of pharynx, pixel size: 11.92 µm.

Figures 20 , 21 , 22

##### Material examined.

Specimen **8922**: unknown collecting site and date. Mislabeled as *G. blaseri*. Three dimensional (3D) images and virtual sections of specimen 8922 were obtained through microcomputed tomography. Subsequently, the following body parts of this specimen were histologically processed: Transverse sections of anterior extremity on 38 slides; horizontal sections of region immediately posterior to anterior extremity on 31 slides; transverse sections of pre-pharyngeal region on 31 slides; transverse sections of posterior extremity on 18 slides; remaining part of body preserved in 80 % ethanol.

##### External aspect.

Fixed worm ~90 mm long, and 2.5 mm wide. Body elongate, with parallel margins; dorsum convex, ventral side flat, and margins rounded. Anterior and posterior extremities blunt (Fig. 20A, C). Dorsum ocher yellow in color, ornamented with five longitudinal stripes of terra brown color, i.e., median stripe bounded on either side by a paramedian stripe, externally to which is a lateral stripe. These stripes are irregularly faded, mainly in first quarter of body. Ventral side ochre yellow in mid-line, 0.9 mm in width, and terra brown laterally.

Single pair of eyes present at 4 mm from anterior extremity, 85 µm in diameter (Fig. 21A). Sensory pits simple invaginations 25–30 µm deep, located marginally in single row. They are distributed between the second and fourth millimeters of the body. Mouth at a distance from the anterior extremity of the body equivalent to 29 % of body length.

##### Internal morphology.

Creeping sole comprising 35 % body width (Fig. 21B). Abundant rhabditogen cells producing erythrophil granules pierce ventral and marginal epidermis.

Cutaneous musculature comprises three layers, namely a subepithelial circular layer (5 µm thick), followed by one diagonal layer with decussate fibers (10 µm thick), and then a well-developed innermost longitudinal layer (70 µm thick dorsally, 75 µm ventrally). Muscle fibers of the longitudinal layer are arranged into bundles of 21–54 fibers. Cutaneous musculature thickness relative to body height in the pre-pharyngeal region, 18 % (Fig. 21A, B).

Five parenchymal muscle layers present: a dorsal layer (20 µm thick) of decussate diagonal fibers, a supraintestinal layer (40 µm thick) of transverse fibers, a subintestinal layer (30 µm thick) of transverse fibers, a subneural layer (40 µm thick) of longitudinal fibers, and a ventral layer (40 µm thick) of decussate diagonal fibers located inside the ventral cutaneous nerve net (Fig. 21C, D).

Central nerve system formed by a pair of ganglia (Fig. 21A) and a set of nerve fibers with the typical form of a rope ladder (Fig. 21B).

Mouth situated at a distance from the root of the pharynx equivalent to 50 % of the pharyngeal pocket length. Pharynx cylindrical, not folded. Esophagus 0.5 mm in length. Pharynx at a distance from the anterior extremity of the body equivalent to 29 % of the body length (Fig. 22). Intestine typical of triclads, consisting of an anterior main branch and two posterior ones, all of them also branched (Fig. 20B). Reproductive organs not developed.

##### Remarks.

It is evident that the external aspect of specimen 8922 does not agree with the original description and figure of the single type specimen of *Ps. blaseri* (Fig. 19). The original description reads: “Length: 160 mm. Width: 20 mm. Dorsum almost completely black, in just a few places it shows the ground color. Pharyngeal and gonopore openings are evident on the ventral side, at 95 mm and 110 mm from anterior extremity, respectively. Ventral side irregularly patterned with marble (so distinguishing from *G. rufiventris*)” (original in Portuguese). Schirch’s description and figure of *Ps. blaseri* match species of *Obama* (Geoplaninae), but every morphological aspect of specimen 8922 mismatches Schirch’s description. Furthermore, there is no individual resembling the original description of *Ps. blaseri* in Schirch’s collection. Thus, the type material, i.e., the holotype, of *Ps. blaseri* should be considered lost.

Specimen 8922 agrees with the diagnosis of *Dolichoplana* (Rhynchodemidae): body shape and size, position of mouth, number of eyes, and thickness of cutaneous longitudinal muscle (Moseley 1877, Graff 1899, Hyman 1940). However, the diagnosis of *Dolichoplana* includes: “external longitudinal muscular bundles very much developed all over the body, but especially in the dorsal regions, where they are the only longitudinal muscles present” (Moseley 1877); an attribute that specimen 8992 does not fit. However, the thin muscle layer, such as the circular and diagonal cutaneous muscles, are frequently only discernible in preparations sectioned in planes tangential to the layer. Moseley might have overlooked the dorsal circular and diagonal layers or, simply, his specimen did not possess them. Nonetheless, the diagnosis of *Dolichoplana* should be re-assessed.

#### Pseudogeoplana doederleini

Taxon classificationAnimaliaTricladidaGeoplanidae

(Schirch, 1929)

21FD097B-4549-5BEA-B27B-8A6B9A85A707

Figures 23 , 24

Geoplana doederleini Schirch, 1929: 33. Type Locality: Baixo Guandu, Espírito Santo, Brazil.
Pseudogeoplana doederleini : Ogren and Kawakatsu 1990: 153.

##### Material examined.

Type material. Three syntypes collected by P. Schirch *Coll* in 1917 at Baixo Guandu, State of Espírito Santo, Brazil. We received them on loan with only a label reading 8914. Each syntype was given an additional identification with the letters A, B, and C. Three dimensional (3D) images and virtual sections of syntype MNRJ 211A were obtained through microcomputed tomography. Syntype **MNRJ 211A**: Preserved in 80 % ethanol. Entire body of syntype **MNRJ 211B** was histologically sectioned as follows: transverse sections of anterior extremity on 12 slides; horizontal sections of ovaries on five slides; transverse sections of pre-pharygeal region on nine slides; sagittal sections of pharynx on 17 slides; sagittal sections of post-pharynx on 17 slides. Syntype **MNRJ 211C**: preserved in 80 % ethanol.

##### Description.

**Figure 23.**
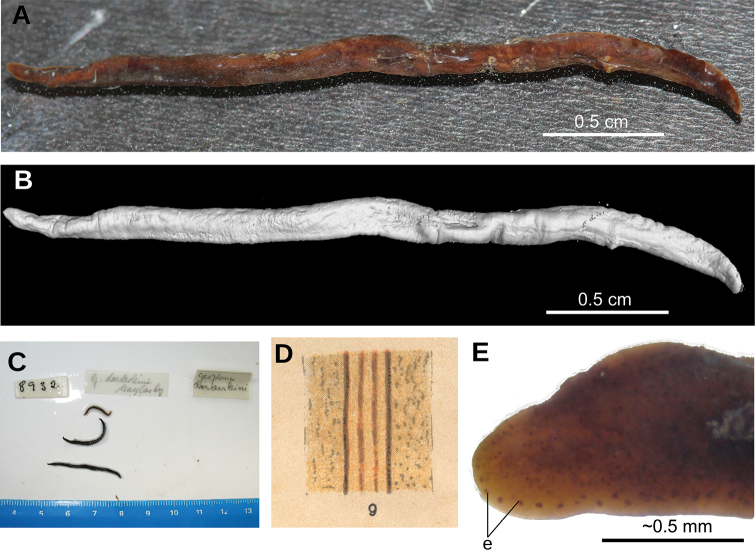
*Pseudogeoplana doederleini* (Schirch 1929) **A** syntype A fixed in dorsal view **B** virtual µCT reconstruction of the body of syntype A **C** three syntypes **D** original illustration of *G. doederleini* by Schirch (1929)**E** anterior extremity of syntype A in clove oil.

**Figure 24.**
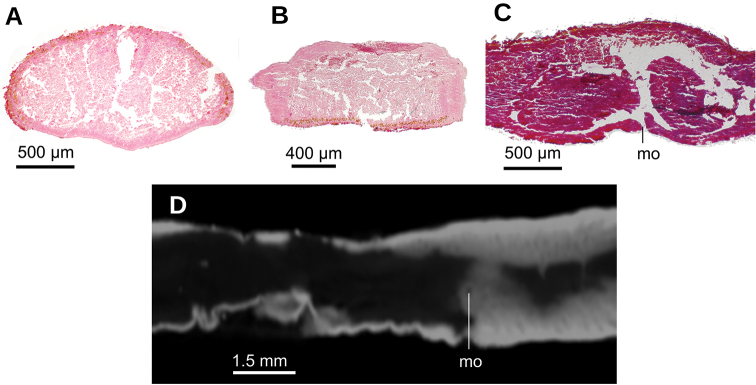
*Pseudogeoplana doederleini* (Schirch 1929). Photomicrographs of syntype B. Sections showing poor quality histological sections. **A** Transverse section of anterior region **B** horizontal section of anterior half of body **C** sagittal section of pharyngeal region **D** virtual sagittal section showing pharyngeal region, pixel size: 5 µm.

All three specimens were rigid and shrunk, with longitudinal cracks (Fig. 23C). Syntype A is 30 mm long, ~3 mm wide (Fig. 23A, B), and 1 mm high; syntype B, 26 mm, 2 mm, and ~0.8 mm, respectively. Elongated body, with parallel margins; anterior and posterior extremities rounded, body sides rounded (Fig. 23A, B, E). Dorsum convex, ventral side convex in some regions, concave in others, the latter probably an effect of dehydration. Ground color of the dorsum beige with yellow olive pigment dots, which are absent in some areas (Fig. 23A). This aspect contrasts with original description and drawing by Schirch (Fig. 23D). Ventral side beige. Eyes monolobate, surround anterior extremity of the body (Fig. 23E). Posterior to this region, eyes were not observed. Relative position of mouth: body length, 61 % (syntype B). Poor quality of the histological and virtual sections (Fig. 24A, D) did not permit the investigation of further morphological details. µCT-derived images did not provide enough contrast for the internal organs and, consequently, we could not obtain any useful virtual section.

##### Remarks.

The body color is somewhat different from the original description (Schirch 1929), since the longitudinal stripes have disappeared after 100 years in preservative. Both the size and form of the body, and the eye distribution pattern agree with the original description. The specimens showed signs of having been accidentally dehydrated (body roughened and rigid) and thus were immersed in Sandison’s solution for five days for tissue rehydration (Sandison 1955). Nonetheless, histological sections of syntype B are of very poor quality, the pharynx and copulatory apparatus are poorly differentiated, and the epithelia are indistinct. µCT derived images are also of very poor quality; the preparation of the specimen for µCT scanning was preceded by the rehydration of the specimen in Sandison’s solution (1955). Even so, the electron-dense contrast agent (PTA) did not penetrate the body and hence the internal organs remained transparent under the X-ray. This failure has been demonstrated by other authors (e.g., Metscher 2009, Fernández et al. 2014). Based on histological sections and µCT derived images of the type material, the species cannot be determined. Therefore, the species is retained in *Pseudogeoplana*.

#### Pseudogeoplana schirchi

Taxon classificationAnimaliaTricladidaGeoplanidae

(Schirch, 1929)

494E275D-1928-51F0-B259-A3F7736F7F10

Figures 25 , 26

Geoplana maximiliani Schirch, 1929: 30; taf. 2, fig, 10. Type locality: Teresópolis, State of Rio de Janeiro, Brazil.
Pseudogeoplana schirchi : Ogren and Kawakatsu 1990: 159. nom. nov.

##### Material examined.

Type material. Single holotype received on loan in 70 % ethanol and, subsequently, three-dimensional (3D) images and virtual sections of syntype MNRJ 217 were obtained by microcomputed tomography. **MNRJ 217**, **holotype**, here designated by monotypy: Teresópolis, State of Rio de Janeiro, Brazil. P. Schirch *Coll* (unknown). Sagittal sections of anterior extremity on 97 slides; horizontal sections of pre-pharyngeal region on 15 slides. Remaining part of body preserved in 80 % ethanol.

##### External aspect.

**Figure 25.**
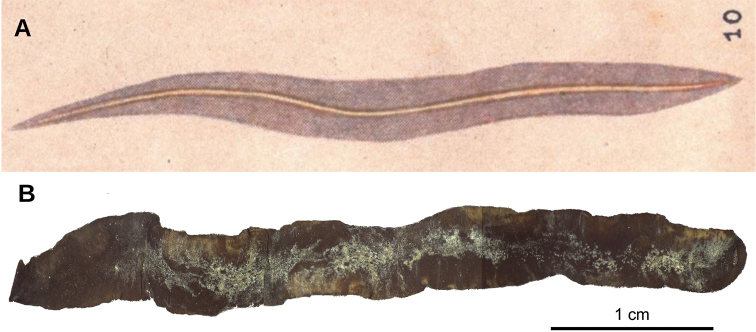
*Pseudogeoplana schirchi* (Schirch 1929), Holotype. **A** Original illustration of *Ps. schirchi* by Schirch (1929)**B** dorsal view of fixed holotype.

Fixed holotype measured 45 mm long, 6 mm wide and 0.9 mm high. Elongated body, with parallel margins; anterior extremity tapers, posterior rounded. Dorsum convex, ventral side flat. Dorsum has yellow olive color, except in some parts where epithelium is lost (Fig. 25B). Ventral side brown grey with some darker regions. Relative position of mouth: body length, 58 %.

##### Internal morphology.

**Figure 26.**
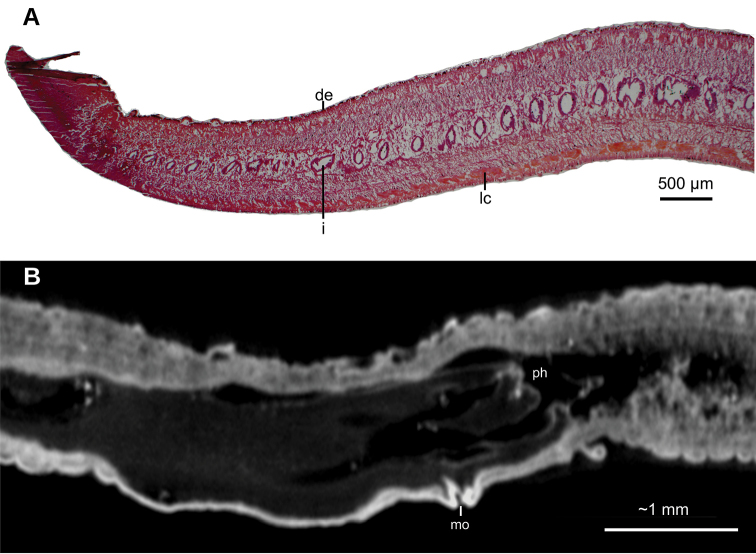
*Pseudogeoplana schirchi* (Schirch 1929). Holotype. **A** Photomicrograph of a sagittal section of anterior extremity **B** virtual sagittal section showing pharyngeal region, pixel size: 9 µm.

Creeping sole comprised ~88 % body width. Histological sections of holotype are of poor quality. Cutaneous musculature comprises three layers: a subepithelial circular layer, followed by a diagonal layer of decussate fibers and a clearly distinguishable innermost longitudinal layer. Muscle fibers of the longitudinal layer (90 µm thick dorsally; 70 µm thick ventrally) arranged into bundles (Fig. 26A). No muscle modifications at the anterior extremity of the body.

Mouth situated at a distance from the root of the pharynx equivalent to 56 % of the pharyngeal pocket length (Fig. 26B). Pharynx cylindrical, occupying ~90 % of pharyngeal pocket. Esophagus, apparently absent. Copulatory apparatus not developed, neither are testes or ovaries.

##### Remarks.

Schirch did not provide any measurement or description of the specimen he studied but illustrated its dorsal aspect (Fig. 25A). The specimen presents parallel body sides, whereas the figure shows a slightly lanceolate specimen. This situation casts some doubts on the labeling (as is the case of *Ps. blaseri*, see above). The specimen was apparently dehydrated for an uncertain period. This might explain the differences in body shape.

Most features diagnosing the subfamily Geoplaninae were observed in the holotype, namely, creeping sole covering most of ventral surface, mouth posterior to mid-body, and a well-developed cutaneous musculature organized into bundles. The immaturity of the specimen makes it impossible to know the position of the testes.

The specimen collected in Teresópolis was identified by Schirch as *Geoplana maximiliani* Schultze & Müller, 1857 (currently placed in *Pseudogeoplana*), originally from Blumenau, State of Santa Catarina, Brazil. Froehlich (1959) considered the shape and color pattern of Schirch’s specimen as compatible with Schultze and Müller’s species, but refrained from confirming its conspecificity because the internal morphology of the specimens from both localities was unknown, plus they were collected far from each other (Froehlich 1959). Ogren and Kawakatsu (1990) endorsed Froehlich’s opinion and gave a new name, namely *Pseudogeoplana schirchi*, to the specimen from Teresópolis to avoid further confusion. As no further morphological details could be observed, the species will remain in *Pseudogeoplana*.

#### Pseudogeoplana wetzeli

Taxon classificationAnimaliaTricladidaGeoplanidae

(Schirch, 1929)

62AE72C9-876F-544D-BB6D-E2FA59A25115

**Figure 27.**
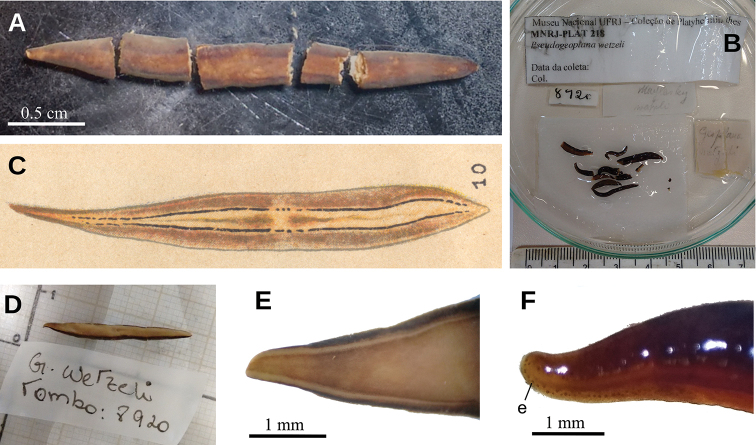
*Pseudogeoplana wetzeli* (Schirch 1929) **A** dorsal view of syntype A in 80 % ethanol after being sectioned for histological processing **B** set of all syntypes with original labels **C** original illustration of *G. wetzeli* by Schirch (1929)**D** ventral view of **s**yntype A in 80 % ethanol on graph paper **E** ventral view of cephalic region of syntype A **F** lateral view of cephalic region of syntype A in clove oil.

**Figure 28.**
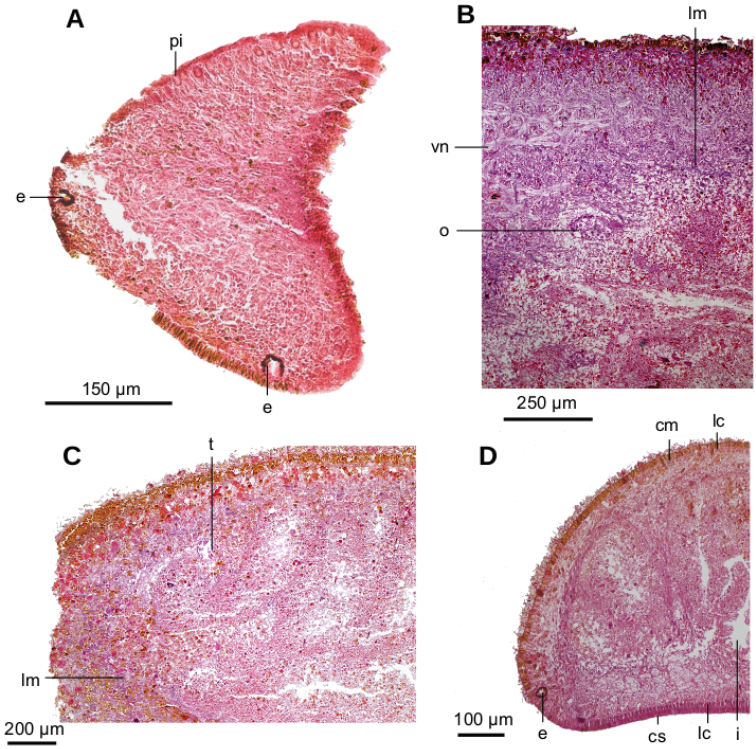
*Pseudogeoplana wetzeli* (Schirch 1929). Photomicrographs of syntype A. **A** Horizontal section of anterior extremity **B** horizontal section of ovarian region **C** horizontal section of testicular region **D** transverse section of anterior extremity.

**Figure 29.**
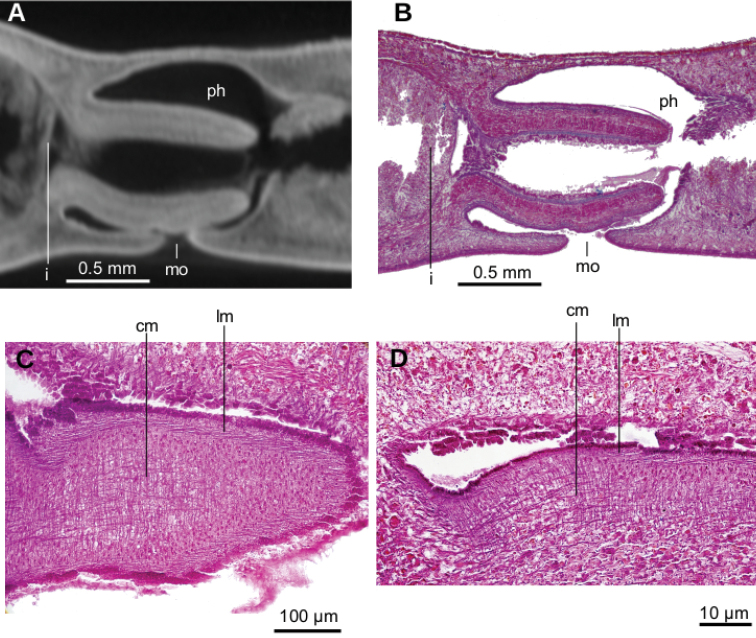
*Pseudogeoplana wetzeli* (Schirch 1929), syntype A. **A** Virtual section of pharynx, pixel size: 4.77 µm **B** photomicrograph of sagittal section of pharynx **C** photomicrograph of tangential section of outer epithelium of pharynx and its muscle, in sagittal plane **D** photomicrograph of sagittal section of outer epithelium of pharynx and its underlying muscle.

Figures 27 , 28 , 29

Geoplana wetzeli Schirch, 1929: 32. Type locality: Baixo Guandu, Espírito Santo, Brazil.
Pseudogeoplana wetzeli : Ogren and Kawakatsu 1990: 160.

##### Material examined

(Fig. 27A, B). Type material. Seven syntypes. Baixo Guandu, Espírito Santo, Brazil. P. Schirch leg., 1917. They were received on loan in 70 % ethanol with only a label reading 218. Each syntype was given an additional identification with a letter, A–G. Parts of the body of syntype **MNRJ 218A** were first studied using µCT followed by traditional histology: transverse sections of anterior extremity on 26 slides; horizontal sections of ovarian region on 29 slides; sagittal sections of pharyngeal region on 33 slides and transverse sections of post-pharyngeal region on 18 slides. Remaining part of body preserved in 80 % ethanol. Following seven syntypes were examined and retained in 80 % ethanol: syntypes **MNRJ 218B**, **C**, **D**, **E**, **F**, **G**. Parts of them were fragmented.

##### External aspect.

Fixed syntype A (Fig. 27A) measured 30 mm long, 3 mm wide, and ~1.2 mm high; other syntypes are smaller (Fig. 27B). Anterior extremity rounded, posterior pointed, body sides rounded. Dorsum convex, ventral side flat. Ground color of the dorsum olive brown with a median band (as wide as 1/3^rd^ of body width) of mahogany brown color that does not reach the extremities of the body (Fig. 27B). Schirch (1929) illustrated the external aspect of the species (Fig. 27C). Ventral side lemon yellow (Fig. 27D, E).

Eyes monolobate, 38 µm in diameter and surround the anterior extremity of the body (Fig. 27F). Posterior to the second millimeter, the eyes spread onto the dorsum and at 4.5 mm occupy the entire dorsum. Sensory pits are simple invaginations 40 µm deep, located ventro-marginally in a single row at least along a body length of ~5 mm (equal to 17 % of the body length, syntype A; Fig. 28A). Relative position of the mouth: body length, ~52 % (syntype A). Gonopore not developed in any syntype.

##### Internal aspect.

The creeping sole comprises 72 % of the body width. Rhabditogen cells and glands producing erythrophil granules pierce the dorsal and marginal epidermis and creeping sole. Ventral epithelium is pierced by a few glands producing fine xanthophil granules. Glandular margin is absent.

Cutaneous musculature comprises three layers typical of the subfamily Geoplaninae. A subepithelial circular layer, followed by a diagonal layer with decussate fibers (2 µm thick dorsally; 2.5 µm thick ventrally) and an innermost longitudinal layer. Muscle fibers of the longitudinal layer (20 µm thick dorsally; 25 µm thick ventrally) are arranged into bundles of 6–19 fibers each. Cutaneous musculature thickness relative to the body height in the post-pharyngeal region, 4.5 %.

Parenchymal musculature formed by a supraintestinal layer of transverse fibers and a subintestinal layer of transverse fibers, both layers interspersed with longitudinal fibers (Fig. 28B, C). Longitudinal parenchymal muscle fibers were best observed in the ovarian region; in the pharyngeal region longitudinal fibers are scarcer. Suboptimal quality of the horizontal sections did not permit us to check whether a third layer, typical of the Geoplaninae (dorsal layer of decussate fibers) is present. In the cephalic region, cutaneous and parenchymal muscle layers are arranged as in the remainder of the body but are less developed. Ventral nerve plate present (Fig. 28B, D).

Mouth situated approximately in the middle of the pharyngeal pouch. Pharynx cylindrical (Fig. 29A, B). Outer pharyngeal musculature consisting of a thin subepithelial longitudinal muscle (5 µm thick), followed by a 25 µm thick circular muscle with longitudinal fibers interspersed (Fig. 29C, D). Lining epithelium of pharyngeal lumen sinuous and ciliated, and underlain by a subepithelial circular muscle (25 µm thick), followed by a longitudinal layer (5 µm). Esophagus absent.

Testes ~40 µm in diameter. They are located dorsally between the supraintestinal parenchymal muscle layer and the intestine, and limited dorsolaterally (Fig. 28C). Anteriormost testes distant from the anterior extremity 5 mm, equivalent to 17 % of the body length; posteriormost testes distant from anterior extremity, i.e. 10.4 mm, equivalent to ~35 % of the body length (syntype A). Incipient sperm ducts only. Copulatory apparatus not developed.

Ovaries are oval, 170 µm in maximum length, and situated immediately above the ventral nerve plate (Fig. 28B). They are located at ~6 mm from the anterior extremity, equivalent to 20 % of the body length (syntype A). Ovovitelline ducts emerge from the dorso-lateral region of ovaries and extend posteriorly only ~200 µm. They contain sperm.

##### Remarks.

Diagnostic features of the Geoplaninae are recognized in this species: creeping sole covering most of ventral surface, mouth posterior to mid-body, well-developed cutaneous musculature organized into bundles, part of the parenchymal muscle layers organized in longitudinal fibers (as they may be present in the subfamily) and testes dorsal (see Carbayo et al. 2013, Almeida et al. 2019).

Although the largest specimen exhibits both male and female gonads, even sperm in ovovitelline ducts, the copulatory apparatus is lacking, the absence of which hinders searching for the taxonomic affinities of this species within the Geoplaninae.

An uncommon feature within this subfamily, the parenchymal longitudinal muscle fibers, might shed some light on the genus to which this species belong. These parenchymal fibers are only known from species of *Geoplana* and *Imbira*. However, *Geoplana* is excluded, since in species of this genus, eyes in the cephalic region are cone-shaped, whereas in *Ps. wetzeli* they are rounded. Regarding the longitudinal parenchymal musculature, the diagnosis of *Imbira* does not exclude *Ps. wetzeli*, but in *Imbira*, the eyes are distributed only marginally (vs. dorsal in *Ps. wetzeli*). In view of the lack of knowledge on the morphology of this species, it should remain in the collective genus *Pseudogeoplana*.

## Discussion

In this paper, we have revised the taxonomy of seven species of land flatworms through the study of the type specimens and additional individuals. The type specimens, considered lost for over eight decades, were deposited in the renowned MNRJ. Over recent years the Museum suffered budget cuts by the Federal government and had no fire protection when in year 2018, a fire turned treasured scientific and cultural collections into ashes (Escobar 2018). It was a double stroke of luck that the types herein studied were on loan when the Museum caught fire and that the part of the building housing part of the collection of invertebrates, survived the fire. As a consequence of the survival of the types, we were successful in studying some of them using virtual sections, i.e., without destroying them. In particular we managed to redescribe *Pa. bresslaui* almost to modern standards from only µCT-derived images, thus preserving the integrity of this precious holotype.

This paper is a step beyond Carbayo et al. (2016) regarding the exploration of the potential of this equipment. Carbayo et al. (2016) discussed the virtues and limitations of exploring µCT equipment for the morphological study of flatworms. The main problems they faced were derived from the limitations of the µCT instrument with regard to the differentiation of small structures. Visualization of such structures depends on two factors, namely the specific limitations of the instrument and the width of the specimen. Carbayo et al. (2016) scanned a wide specimen of *O. otavioi*Carbayo et al., 2016 with an instrument that produces images with a spatial resolution that depends on the distance between the specimen and the source of X-rays; the higher the magnification intended, the shorter the distance required. Therefore, the specimen was scanned only at 6.04 µm, whereas the scan can provide <4 µm resolution (https://www.bruker.com/pt/products/microtomography/micro-ct-for-sample-scanning/skyscan-1273/technical-details.html). Zeiss Xradia Versa XRM-510 overcame this problem through a scintillator that converts X-rays to visible light. Subsequently, this light is optically magnified (https://www.zeiss.com/microscopy/int/products/x-ray-microscopy/zeiss-xradia-510-versa.html).

The highest resolution of this instrument reaches 0.7 µm. We used this instrument to scan the holotype of *Pa. bresslaui*. Because this specimen is narrow, we were able to scan regions of interest (prostatic vesicle and cephalic extremity) at resolutions as small as 1.9 µm. This was possible even though the cephalic extremity is bent, so doubling its effective width to ~10 mm (see Fig. 5A, C, E). Consequently, we could clearly reveal the eyes, sensory pits and muscle bundles with the µCT instrument. However, minute structures, such as single muscle fibers, cilia, and cell nuclei remained indiscernible. This is why we did not provide the width of the creeping sole, the organization of the parenchymal muscle systems, and details of the epithelial lining the female atrium of the holotype.

As expected, secretions with acidic-basic nature also remained elusive. This is another limitation of µCT instruments. X-ray based equipment produces images of electron-dense objects with different levels of grey. Contrast enhancer agents, such as PTA are not tissue-specific (see Metscher 2009a and b) and seemingly link to secretions independently of its acidic nature. Revealing the nature of the secretions is important in functional histology (e.g. Winsor 1998, Cima 2017). Nonetheless, in rare occasions, this feature has been used in the taxonomy of land planarians; mainly the nature of glands constituting the glandular margin, although this was only one among many diagnostic characteristics for the species (see Álvarez-Presas et al. 2015).

A promising step in the exploration of the capabilities of µCT instruments will be the use of equipment with a <0.5 µm spatial resolution, although limited to the study of relatively small specimens (Shearer et al. 2016). Also, this technique does not prevent further and future approaches, since phosphotungstic acid does not cause tissue damage or changes at a cellular level (Faulwetter et al. 2013), and µCT X-rays do not fragment DNA (Paredes et al. 2012).

## Supplementary Material

XML Treatment for Obama itatiayana

XML Treatment for Paraba bresslaui

XML Treatment for Pasipha plana

XML Treatment for Pseudogeoplana arpi

XML Treatment for Pseudogeoplana blaseri

XML Treatment for Dolichoplana

XML Treatment for Pseudogeoplana doederleini

XML Treatment for Pseudogeoplana schirchi

XML Treatment for Pseudogeoplana wetzeli
